# Patterns of cerebral damage in multiple sclerosis and aquaporin-4 antibody-positive neuromyelitis optica spectrum disorders—major differences revealed by non-conventional imaging

**DOI:** 10.1093/braincomms/fcae295

**Published:** 2024-08-30

**Authors:** Paweł Jakuszyk, Aleksandra Podlecka-Piętowska, Bartosz Kossowski, Monika Nojszewska, Beata Zakrzewska-Pniewska, Maciej Juryńczyk

**Affiliations:** Laboratory of Brain Imaging, Polish Academy of Sciences, Nencki Institute of Experimental Biology, 02-093 Warsaw, Poland; Department of Neurology, Medical University of Warsaw, 02-091 Warsaw, Poland; Laboratory of Brain Imaging, Polish Academy of Sciences, Nencki Institute of Experimental Biology, 02-093 Warsaw, Poland; Department of Neurology, Medical University of Warsaw, 02-091 Warsaw, Poland; Department of Neurology, Medical University of Warsaw, 02-091 Warsaw, Poland; Laboratory of Brain Imaging, Polish Academy of Sciences, Nencki Institute of Experimental Biology, 02-093 Warsaw, Poland

**Keywords:** NMOSD, magnetic resonance imaging, tractometry analysis, white matter damage, differential diagnosis

## Abstract

Multiple sclerosis and aquaporin-4 antibody neuromyelitis optica spectrum disorders are distinct autoimmune CNS disorders with overlapping clinical features but differing pathology. Multiple sclerosis is primarily a demyelinating disease with the presence of widespread axonal damage, while neuromyelitis optica spectrum disorders is characterized by astrocyte injury with secondary demyelination. Diagnosis is typically based on lesion characteristics observed on standard MRI imaging and antibody testing but can be challenging in patients with in-between clinical presentations. Non-conventional MRI techniques can provide valuable diagnostic information by measuring disease processes at the microstructural level. We used non-conventional MRI to measure markers of axonal loss in specific white matter tracts in multiple sclerosis and neuromyelitis optica spectrum disorders, depending on their relationship with focal lesions. Patients with relapsing-remitting multiple sclerosis (*n* = 20), aquaporin-4 antibody-associated neuromyelitis optica spectrum disorders (*n* = 20) and healthy controls (*n* = 20) underwent a 3T brain MRI, including T_1_-, T_2_- and diffusion-weighted sequences, quantitative susceptibility mapping and phase-sensitive inversion recovery sequence. Tractometry was used to differentiate tract fibres traversing through white matter lesions from those that did not. Neurite density index was assessed using neurite orientation dispersion and density imaging model. Cortical damage was evaluated using T_1_ relaxation rates. Cortical lesions and paramagnetic rim lesions were identified using phase-sensitive inversion recovery and quantitative susceptibility mapping. In tracts traversing lesions, only one out of 50 tracts showed a decreased neurite density index in multiple sclerosis compared with neuromyelitis optica spectrum disorders. Among 50 tracts not traversing lesions, six showed reduced neurite density in multiple sclerosis (including three in the cerebellum and brainstem) compared to neuromyelitis optica spectrum disorders. In multiple sclerosis, reduced neurite density was found in the majority of fibres traversing (40/50) and not traversing (37/50) white matter lesions when compared to healthy controls. A negative correlation between neurite density in lesion-free fibres and cortical lesions, but not paramagnetic rim lesions, was observed in multiple sclerosis (39/50 tracts). In neuromyelitis optica spectrum disorders compared to healthy controls, decreased neurite density was observed in a subset of fibres traversing white matter lesions, but not in lesion-free fibres. In conclusion, we identified significant differences between multiple sclerosis and neuromyelitis optica spectrum disorders corresponding to their distinct pathologies. Specifically, in multiple sclerosis, neurite density reduction was widespread across fibres, regardless of their relationship to white matter lesions, while in neuromyelitis optica spectrum disorders, this reduction was limited to fibres passing through white matter lesions. Further studies are needed to evaluate the discriminatory potential of neurite density measures in white matter tracts for differentiating multiple sclerosis from neuromyelitis optica spectrum disorders.

## Teaser

Jakuszyk *et al*. report that a decrease in neurite density index measured by magnetic resonance imaging is observed in white matter tracts in multiple sclerosis regardless of whether tracts traverse or not traverse white matter lesions, while in neuromyelitis optica spectrum disorders, it is only limited to tracts traversing through lesions.

## Graphical Abstract

**Graphical Abstract:**
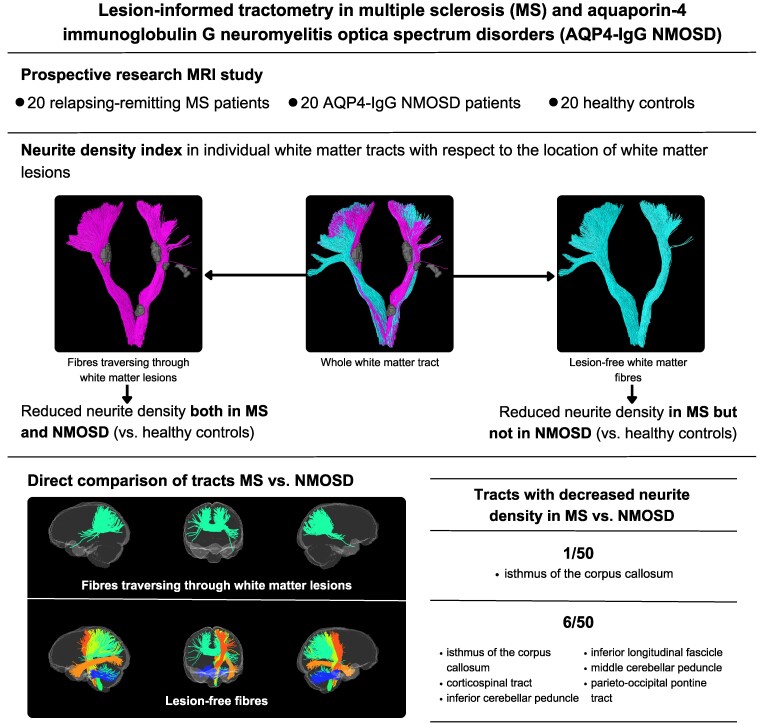

## Introduction

Multiple sclerosis and neuromyelitis optica spectrum disorders (NMOSD) are distinct autoimmune diseases of the CNS characterized by acute attacks of the optic nerve, spinal cord and brain/brainstem inflammation. In the majority of patients, NMOSD is associated with the presence of autoantibodies against aquaporin-4 (AQP4) expressed on astrocytes, while multiple sclerosis is considered to be driven by cell-mediated autoreactivity against myelin peptides.^1^ Due to overlapping symptomatology, the differential diagnosis may be challenging but is crucial given distinct treatment and prognosis in both diseases. Conventional MRI of the brain and/or the spinal cord is the most useful early diagnostic tool, as it helps guide clinicians towards the most likely diagnosis based on well-established discriminators, including longitudinally extensive spinal cord lesions, which is a hallmark of NMOSD, and brain lesions suggestive of multiple sclerosis, such as Dawson’s fingers or curved juxtacortical lesions.^2,3^ These features, however, are less useful in patients with few brain or short cord lesions or in cases of in-between appearances. An assay detecting AQP4 antibodies in the patient serum is the gold standard for the diagnosis of NMOSD but is hardly available outside of specialist centres, takes days to perform and report, and if negative does not completely exclude NMOSD (‘true’ seronegative NMOSD, false negativity on suboptimal testing, such as enzyme-linked immunoabsorbent assay, false negativity on optimal testing following immunosuppression or with undetectable antibody before seroconversion).^4-6^ Misdiagnosis of multiple sclerosis and NMOSD still occurs and there is a major need for easily available, non-invasive and accurate diagnostic tools.^7^

Although multiple sclerosis and AQP4 antibody NMOSD may present with overlapping clinical and radiological features, their pathology is distinct.^8,9^ In particular, multiple sclerosis is defined as a primarily demyelinating disease, while AQP4 antibody NMOSD is an astrocytopathy with secondary damage to oligodendrocytes and neurons related to disease attacks. In contrast to AQP4 antibody NMOSD, multiple sclerosis is characterized by progressive neurodegeneration, which clinically translates into disability accumulation independent of relapses, a feature considered highly atypical of NMOSD.^1^ Diffuse neurodegeneration in multiple sclerosis is secondary to neuronal and axonal damage within focal lesions (through anterograde and retrograde degeneration), including chronic active lesions characterized by inflammation at the lesion edge considered to be particularly destructive in this regard.^10,11^ Interestingly, however, axonal loss in normal-appearing white matter (NAWM) in multiple sclerosis may also develop independently of focal lesions and in such cases was shown to correlate with cortical damage.^12^

Non-conventional MRI is a promising diagnostic tool in CNS diseases as it allows for the assessment of pathological processes at the microstructural level, also in areas which appear normal on conventional imaging. These techniques are increasingly sensitive towards white matter damage, including biophysical modelling of diffusion data using neurite orientation dispersion and density imaging (NODDI), likely providing a more sensitive measure of axon integrity when compared to diffusion tensor imaging (DTI) or magnetization transfer ratio (MTR).^13^ Importantly, previous studies implementing DTI and/or MTR demonstrated diffuse damage in the white matter outside of hyperintense lesions seen on T_2_-weighted images (NAWM) in multiple sclerosis.^14,15^ Whether this observation also applies to NMOSD is unclear. Some studies indeed reported global NAWM damage in NMOSD similar to multiple sclerosis,^16,17^ but others showed damage only in selected tracts, including optic radiation and/or corticospinal tracts,^18,19^ or did not identify any damage in NAWM at all.^20^

We have hypothesized that novel MRI techniques allowing for the assessment of neurite density in individual tracts may identify significant differences between multiple sclerosis and NMOSD corresponding to their distinct pathology. In particular, since both diseases display axonal damage within disease lesions,^8,10^ we aimed to study axonal loss independent of white matter lesions suspecting it might be present in multiple sclerosis characterized by diffuse neurodegeneration but not in NMOSD. To address this hypothesis, we have designed and undertaken a 3T prospective MRI research study where we identified using MRI tractography tracts traversing through white matter lesions and those that were lesion-free both in multiple sclerosis and NMOSD in order to measure neurite density (NODDI model).^21^ In the second step we aimed to relate our findings to the presence of chronic active lesions (identified as paramagnetic rim lesions, PRLs, on MRI) and cortical demyelination (measured by T_1_ relaxation rates and the presence of cortical lesions), which have been both pathologically linked to diffuse neurodegeneration in multiple sclerosis.^22,23^

## Materials and methods

### Participants

This prospective cohort study was approved by the bioethics committee at the Institute of Psychiatry and Neurology in Warsaw, Poland (nr 8/2021). Written informed consent was obtained from all participants. Multiple sclerosis patients (*n* = 20) were recruited from a disease-modifying treatment clinic in the Department of Neurology, Wolski Hospital, Warsaw (MJ). NMOSD patients (*n* = 20) were recruited from NMOSD clinics in Wolski Hospital (MJ) and the Department of Neurology, Warsaw Medical University (A.P.-P., M.N. and B.Z.-P.). All multiple sclerosis patients fulfilled revised McDonald criteria^24^ for relapsing-remitting multiple sclerosis. NMOSD patients fulfilled the revised 2015 NMOSD criteria^25^ and were all AQP4-IgG-positive on a fixed cell-based assay. All multiple sclerosis and NMOSD patients were scanned at least two months after the last disease attack. Healthy controls (HC) (*n* = 20) sex- and age-matched to the NMOSD cohort were recruited via social media channels at the Laboratory of Brain Imaging, Nencki Institute of Experimental Biology. All participants were recruited between September 2021 and July 2022.

### Clinical data

Multiple sclerosis and NMOSD patients’ clinical data were obtained from clinical files supplied by treating neurologists from referring centres, and included onset attack presentation, attack phenotype, Expanded Disability Status Scale (EDSS) at study scan time and the present disease-modifying therapy.

### MRI image acquisition

All study participants were scanned in the Laboratory of Brain Imaging, Nencki Institute of Experimental Biology, Polish Academy of Sciences in Warsaw. MRI data were acquired using a 3T Siemens Trio scanner (Siemens Erlangen, Germany) with a 32-channel array head coil. The MRI protocols included: (i) T_1_-weighted magnetization-prepared rapid acquisition gradient echo (MPRAGE): repetition time/echo time/inversion time = 2530/3.32/1100 ms, acquisition time = 6 min; (ii) fluid-attenuated inversion recovery (FLAIR): repetition time/echo time/inversion time = 5000/388/2100 ms, both with 1 mm^3^ isotropic spatial resolution, acquisition time = 7 min; (iii) multi-shell diffusion-weighted sequence (DWI): repetition time/echo time/resolution = 3660/101 ms/2 × 2 × 2 mm^3^ isotropic with b-values of 0/500/1250/2500 s/mm^2^ and 13/18/36/53 measurements per shell, respectively, and an additional diffusion acquisition with seven measurements of *b*-value 0 s/mm^2^ with reversed phase encoding direction to correct for susceptibility-induced distortions, acquisition time = 8 min; (iv) T_1_-weighted MPRAGE with two separate readouts at different inversion times (MP2RAGE): repetition time/echo time/inversion time 1/inversion time 2 = 5000/2.96/700/2500 ms, and 1 mm^3^ isotropic spatial resolution, acquisition time = 8 min; (v) T_2_*-weighted gradient echo, multi-echo quantitative susceptibility mapping (QSM) sequence with repetition time/echo times = 27/4.65, 9.15, 13.65, 18.15, 18.15, 22.65 ms and 0.7 × 0.7 × 1.0 mm^3^ spatial resolution, acquisition time = 8 min; (vi) Phase-sensitive inversion recovery (PSIR) sequence: repetition time/echo time/inversion time = 4000/381/380 ms, with 0.9 mm^3^ isotropic spatial resolution, acquisition time = 6 min. The whole scanning session lasted ∼43 min. Due to technical difficulties, the MP2RAGE sequence could not be acquired for three multiple sclerosis patients, and the QSM sequence was not obtained for two HC and one multiple sclerosis patient.

### NODDI model estimation

DWI pre-processing followed the MRtrix3 pipeline.^26^ EDDY QC framework was used to assess data quality.^27^ Neurite density index (NDI) maps were generated by fitting the nonlinear three-compartment NODDI model^28^ to preprocessed DWI data using the NODDI Matlab Toolbox.

### Lesion segmentation and volume estimation

Lesion segmentation was done by manually tracing hyperintense white matter lesions on FLAIR images using FSLeyes software.^29^ Created binary lesion masks were then registered to MPRAGE images using a rigid body registration algorithm. Subsequently, the FSL’s lesion-filling utility was used to estimate intensities from surrounding white matter and fill signal dropouts in T_1_-weighted images improving the registration and segmentation of the MPRAGE images, which were used to create registration matrices to DWI space. At each stage of the registration process, the datasets were visually reviewed to ensure the quality of the analysis. The T_2_-hyperintense white matter lesion volume was determined for each patient based on their lesion masks. To account for variations in intracranial volume, the lesion volume was normalized by dividing it by the estimated total intracranial volume (ETIV) obtained using Freesurfer. The resulting value was then multiplied by 100 to represent a percentage of the ETIV.

In order to assess differences in lesion burden between individual tracts in multiple sclerosis and NMOSD, bundle load (defined for a given tract as the volume of streamlines that traverse lesions divided by the total volume of that tract-of-interest) in each of the 50 investigated tracts was computed.^30^

The number of cortical lesions was determined through visual inspection of PSIR and the corresponding FLAIR images, following the guidelines outlined by Filippi *et al.*^31^

### Tractography and tractometry

Two shells (*b* = 0, 2500 s/mm^2^) were extracted from the DWI datasets. Response functions for single-fibre white matter, as well as grey matter and cerebrospinal fluid, were estimated from the data using an unsupervised algorithm.^32^ Single-Shell 3-Tissue Constrained Spherical Deconvolution (SS3T-CSD) was performed to obtain fibre orientation distributions for white matter. For every subject, 50 white matter tracts were generated with TractSeg,^33^ which used the distribution peaks data obtained from the SS3T-CSD algorithm as input to generate the tracts. Five thousand streamlines, approximating fibre bundles, were computed for each tract. To exclude the effect of lesions on the reconstruction of physiological white matter tracts, the accuracy of tract segmentation performed by TractSeg was visually inspected by the study investigator (PJ) for any anomalies but no problems have been identified. To segment the tracts into fibres traversing and not traversing through white matter lesions the binary lesion masks were supplied to the MRtrix3 tckedit command, which discarded all streamlines that entered any region from the lesion mask or conversely only included streamlines traversing through the lesions.

The NDI values were obtained with TractSeg’s tractometry module.^34^ The algorithm calculated a central line for each tract and divided the tract’s streamlines into 100 segments. Values from the NDI maps were sampled on each segment and pooled to obtain one NDI value per tract. NDI values were obtained for unsegmented tracts as well as for tract fibres that traversed and did not traverse through white matter lesions. To exclude focally decreased NDI values within white matter lesions^35^ from the tract fibres that traversed through them, we employed an in-house algorithm to identify lesion locations along each tract. Subsequently, NDI values associated with focal lesion points were removed.

### Assessment of T_1_ relaxation rates in cerebral cortex

T_1_ relaxation rates for the cerebral cortex were acquired through MP2RAGE sequences. Cortical segmentation was performed using Freesurfer’s segmentation on the standard T_1_-weighted MPRAGE image. The MP2RAGE T_1_ relaxation maps were registered to Freesurfer’s reconstruction using the previously denoised UNIFIED images.^36^ Ciftify tool from Human Connectome Project^37^ was used to create grey matter ribbon and project voxels with coordinates located between the white and pial surfaces to sample and smooth values of T_1_ relaxation rates. Then averaged estimates were extracted for the Destrieux atlas, which comprises 150 regions of the cerebral cortex.^38^

### PRLs classification

QSM maps were calculated from the multi-echo magnitude and phase T_2_*-weighted images with SEPIA Matlab toolbox. Morphology enabled dipole inversion algorithm^39^ was used for unwrapping phase data and estimating susceptibility maps with the cerebrospinal fluid used for value referencing. The QSM images were visually inspected for PRLs and cross referenced with corresponding FLAIR images (Supplementary Fig. 1).

### Statistical analysis

**Figure 1:**
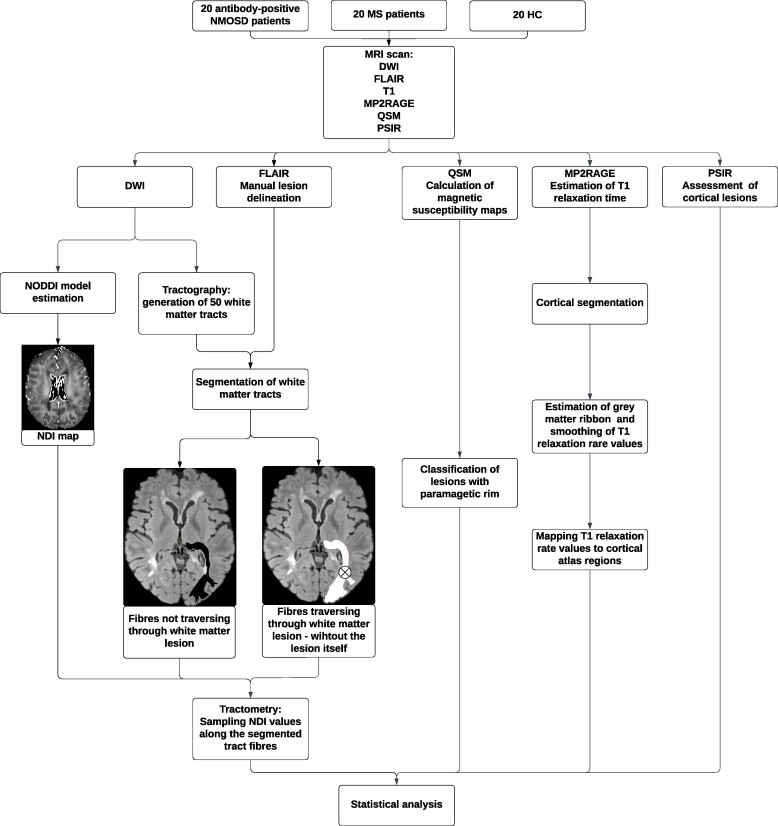
**Analysis workflow.** Figure illustrates the most important steps of the analysis process. Optic radiation is shown as an example of a segmented white matter tract—white area represents fibres traversing through a white matter lesion (depicted as a crossed circle), while black area represents lesion-free fibres. NMOSD, neuromyelitis optica spectrum disorders; MS, multiple sclerosis; HC, healthy controls; NODDI, neurite orientation dispersion and density imaging; NDI, neurite density index; EDSS, Expanded Disability Status Scale; FLAIR, fluid-attenuated inversion recovery; MP2RAGE, magnetization-prepared 2 rapid acquisition gradient echo; DWI, diffusion-weighted imaging; QSM, quantitative susceptibility mapping; PSIR, phase-sensitive inversion recovery.

Normality and equality of variance were tested for all variables. Cohen’s d (*d*) was used as an effect size measure. Differences in demographic and disease characteristics were assessed with one-way ANOVA, Welch’s *t*-test and Mann–Whitney *U* test. Welch’s *t*-test statistics were calculated for each tract to assess between-group differences. Spearman’s rank correlation was used to study the relationship between lesion count (PRLs, cortical lesions) and NDI in individual tracts. For all hypotheses containing multiple comparisons (multiple tracts or cortical regions), the familywise error was controlled for with Benjamini–Hochberg correction (*P* = 0.05). To visualize the analysis workflow, we present the process in Fig. 1.

## Results

### Sample demographics

Female to male ratio was 18:2 in each study group (Table 1). At the study scan, multiple sclerosis patients were younger (*M =* 35.4, SD = 9.4 years old) when compared with both AQP4 antibody NMOSD patients (*M =* 50, SD = 13.2) and HC (*M =* 48.8, SD = 7.9, *P* < 0.01). Multiple sclerosis patients had significantly lower EDSS compared to NMOSD patients (*M =* 2.1, SD = 2.2 versus *M =* 3.8, SD = 1.9, *P* = 0.01). There was no statistically significant difference in disease duration or time from the last attack to study MRI scan between multiple sclerosis and NMOSD (Table 1).

**Table 1:** Demographics and MRI characteristics of patients and controls

	RRMS	AQP4-IgG + NMOSD	HC
Sex, *n* (male/female)	20 (2/18)	20 (2/18)	20 (2/18)
Age, years, mean ± SD	35 ± 9***	50 ± 13	49 ± 8
EDSS score, median (range)	1 (0–6.5)**	3.7 (1–7.5)	
Disease duration years, mean ± SD	4.7 ± 5.5	8.4 ± 7.5	
Time from last attack to study scan, mean ± SD (months)	18.8 ± 34.7	35.9 ± 29.5	
Brain lesions present, *n*	20	17	
Normalized white matter lesion volume (%), mean ± SD	0.69 ± 0.86**	0.23 ± 0.36	
PRL count, mean ± SD	1.1 ± 1.7		
Cortical lesions count, mean ± SD	7.8 ± 12.5*	0.45 ± 1.2	
Disease-modifying therapy, (*n*)	Dimethyl fumarate (11) Glatiramer acetate (1) Interferon-beta (2) Ocrelizumab (2) Untreated (4)	Azathioprine (2) Azathioprine + Methylprednisolone (1) Azathioprine + Prednisolone (1) Inebilizumab (3) Intravenous immunoglobulin (1) Methotrexate + Methylprednisolone (1) Prednisolone (3) Rituximab (4) Satralizumab (1) Satralizumab + Azathioprine (3)	
Number of patients who had previous TM, *n*	11	18	

^*^ *P* < 0.05, **P < 0.01, ***P < 0.001; NMOSD, neuromyelitis optica spectrum disorders; RRMS, relapsing-remitting multiple sclerosis; HC, healthy controls; *n*, number of participants; SD, standard deviation; %, percentage; TM, transverse myelitis; AQP4-IgG+, aquaporin-4 immunoglobulin G positive; EDSS, Expanded Disability Status Scale; PRL, paramagnetic rim lesions.

### Comparison of MRI features between groups

**Figure 2:**
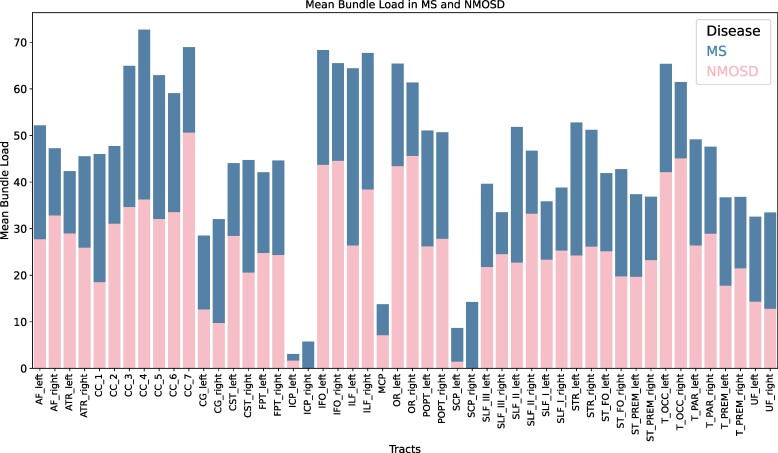
**Visualzation of mean lesion burden in multiple sclerosis (blue, higher values) and NMOSD (pink, lower values).** NMOSD bundle load bar is overlaid on the multiple sclerosis bundle load bar. *Y*-axis represents the percentage of fibres in each tract with lesion involvement. Mean bundle load as assessed by Welch’s *t*-tests did not significantly differ between diseases in any of the investigated white matter tracts (*P* > 0.05). NMOSD, neuromyelitis optica spectrum disorders; MS, multiple sclerosis; AF, arcuate fascicle; ATR, anterior thalamic radiation; CC, corpus callosum; CC_1, rostrum; CC_2, genu; CC_3, rostral body; CC_4, anterior midbody; CC_5, posterior midbody; CC_6, isthmus; CC_7, splenium; CG, cingulum; CST, corticospinal tract; FPT, fronto-pontine tract; ICP, inferior cerebellar peduncle; IFO, inferior occipito-frontal fascicle; ILF, inferior longitudinal fascicle; MCP, middle cerebellar peduncle; OR, optic radiation; POPT, parieto-occipital pontine tract; SCP, superior cerebellar peduncle; SLF_I, superior longitudinal fascicle I; SLF_II, superior longitudinal fascicle II; SLF_III, superior longitudinal fascicle III; STR, superior thalamic radiation; ST_FO, striato-fronto-orbital; ST_PREM, striato-premotor; T_OCC, thalamo-occipital; T_PAR, thalamo-parietal; T_PREM, thalamo-premotor; UF, uncinate fascicle.

Multiple sclerosis patients had higher white matter lesion volume when compared with NMOSD patients (*M =* 0.69, SD = 0.86 versus *M =* 0.23, SD = 0.36, *P* = 0.04, Table 1). Three NMOSD patients did not have any brain lesions. In NMOSD patients two white matter tracts did not have any lesion load in any patient (right inferior cerebellar peduncle and right superior cerebellar peduncle). However, no statistically significant difference in bundle load was identified in any white matter tract between multiple sclerosis and NMOSD (*P* > 0.05 for each tract, Fig. 2). Multiple sclerosis patients had significantly more cortical lesions than NMOSD patients (*M =* 7.8, SD = 12.5 versus *M =* 0.45, SD = 1.2, *P* = 0.02). At least one PRL was identified in seven multiple sclerosis patients (between 1 and 5 PRLs per patient) and in none of NMOSD patients.

### NDI differences between MS and AQP4 antibody-positive NMOSD

When assessing unsegmented white matter tracts 17 of 50 tracts were found to have significantly lower NDI values in multiple sclerosis when compared to NMOSD (detailed statistics in Supplementary Table 1).

**Figure 3:**
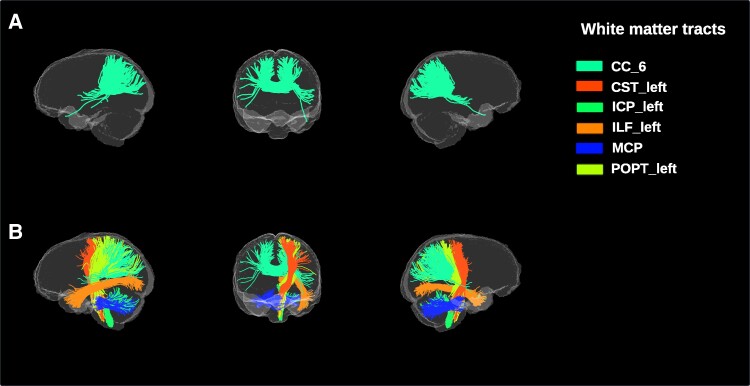
**Visualization of NDI differences between multiple sclerosis and NMOSD.** Significant differences in mean NDI (Welch’s *t*-test). (**A**) White matter fibres traversing through white matter lesions showing significantly lower NDI values in multiple sclerosis patients when compared with NMOSD patients [CC_6, *t*(31.66) = −2.56, *P* = 0.04]. (**B**) White matter fibres not traversing through white matter lesions showing significantly lower NDI values in multiple sclerosis patients when compared with NMOSD patients [CC_6, *t*(37.73) = −2.70, *P* = 0.04; CST_left, *t*(37.98) = −3.08, *P* = 0.02; ICP_left, *t*(36.14) = −3.40, *P* = 0.01; ILF_left, *t*(34.90) = −2.99, *P* = 0.02; MCP, *t*(31.67) = −3.11, *P* = 0.02; POPT_left, *t*(36.08) = −2.73, *P* = 0.04]. NMOSD, neuromyelitis optica spectrum disorders; MS, multiple sclerosis; CC_6, isthmus; CST, corticospinal tract; ICP, inferior cerebellar peduncle; ILF, inferior longitudinal fascicle; MCP, middle cerebellar peduncle; POPT, parieto-occipital pontine tract.

In white matter fibres traversing through white matter lesions, NDI was decreased in multiple sclerosis in only one out of 50 white matter tracts (isthmus of the corpus callosum) when compared to NMOSD (*M =* 0.58, SD = 0.05 versus *M =* 0.63, SD = 0.04, *P* = 0.04, Fig. 3A, Supplementary Table 1 and Figs. 2 and 3).

**Figure 4:**
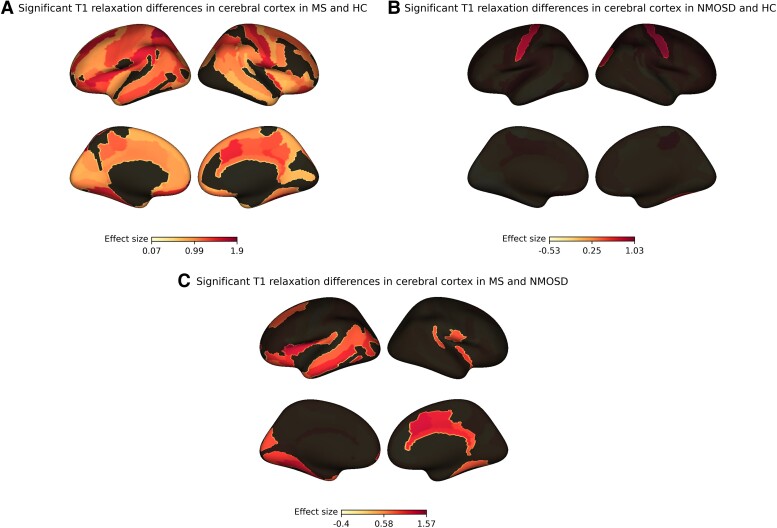
**Visualization of significant between-group differences in T_1_ relaxation rates in cerebral cortex.** Significant differences in mean T_1_ relaxation rates between study groups (Welch’s *t*-test). (**A**) Cortical regions (103/150) showing significantly lower T_1_ relaxation values in multiple sclerosis patients when compared with HC (for detailed statistical values see Supplementary Table 4). (**B**) Cortical regions (4/150) showing significantly lower T_1_ relaxation values in NMOSD patients when compared with HC [right central sulcus, *t*(32.25) = −3.26, *P* = 0.01; left central sulcus *t*(30.37) = −2.84, *P* = 0.03; right superior occipital gyrus, *t*(30.25) = −2.73, *P* = 0.04; right superior occipital sulcus and transverse occipital sulcus, *t*(29.09) = −2.60, *P* < 0.05]. (**C**) Cortical regions (28/150) showing significantly lower T_1_ relaxation values in multiple sclerosis patients when compared with NMOSD patients (for detailed statistical values see Supplementary Table 4). NMOSD, neuromyelitis optica spectrum disorders; MS, multiple sclerosis; HC, healthy controls.

In white matter fibres not traversing through white matter lesions, multiple sclerosis patients had significantly lower NDI values in 6 out of 50 tracts compared to NMOSD patients, including isthmus of the corpus callosum (*M =* 0.58 SD = 0.04 versus *M =* 0.62, SD = 0.05, *P* = 0.04), left corticospinal tract (*M* = 0.67, SD = 0.03 versus *M* = 0.70, SD = 0.03, *P* = 0.02), left inferior cerebellar peduncle (*M =* 0.68, SD = 0.04 versus *M =* 0.72, SD = 0.02, *P* < 0.01), left inferior longitudinal fascicle (*M =* 0.52, SD = 0.03 versus *M =* 0.54, SD = 0.04, *P* = 0.02), middle cerebellar peduncle (*M =* 0.72, SD = 0.03 versus *M =* 0.75, SD = 0.04, *P* = 0.01), left parieto-occipital pontine tract (*M =* 0.64, SD = 0.02 versus *M =* 0.67, SD = 0.03, *P* = 0.04, Fig. 3B, detailed statistics in Supplementary Table 1 and Figs. 4 and 5).

### NDI differences between MS patients and HC

When assessing unsegmented tracts, 48 out of 50 white matter tracts in multiple sclerosis patients showed a significant decrease in NDI when compared with corresponding tracts from HC (Supplementary Table 2).

After tract segmentation according to lesion location, a decrease in NDI was observed in 40/50 fibres traversing and 34/50 not traversing through white matter lesions in multiple sclerosis versus whole tracts from HC (detailed statistics in Supplementary Table 2 and Figs. 2–5).

### NDI differences between AQP4 antibody-positive NMOSD patients and HC

In unsegmented tracts in NMOSD, no significant differences in NDI were found in white matter tracts versus HC (Supplementary Table 3).

In tract fibres traversing through white matter lesions 9 out of 48 white matter tracts in NMOSD patients showed a significant decrease in NDI when compared with corresponding tracts from HC, while no significant differences in NDI were found in lesion-free tracts between NMOSD and HC (Supplementary Table 3 and Figs. 2–5).

### Cortical damage and PRLs relationship with NDI in white matter tracts not traversing through white matter lesions

We then examined the relationship between cortical damage, as assessed by cortical T_1_ relaxation rates and the presence of cortical lesions, and NAWM damage in our multiple sclerosis and AQP4 antibody NMOSD cohorts. Compared to HC, cortical T_1_ relaxation rates were significantly lower in 103 out of 150 cortical regions in multiple sclerosis patients (Fig. 4A) and in 4 out of 150 in NMOSD patients (Fig. 4B). In NMOSD these regions included right central sulcus (*P* < 0.05); left central sulcus (*P* < 0.05); right superior occipital gyrus (*P* < 0.05); right superior occipital sulcus and transverse occipital sulcus (*P* < 0.05). Multiple sclerosis patients had significantly lower T_1_ relaxation rates in 28 out of 150 cortical regions when compared to NMOSD (Supplementary Table 4, Fig. 4C). In multiple sclerosis cortical lesion count had a statistically significant negative correlation with NDI in 38/50 white matter tracts not traversing through white matter lesions (Supplementary Table 5). No significant correlation between PRLs and NDI was identified in multiple sclerosis in any tract (data not shown).

## Discussion

In this prospective research MRI study, we have implemented advanced imaging techniques to examine damage in the NAWM in multiple sclerosis and AQP4-IgG-positive NMOSD, as compared to cerebral white matter from HC. In particular, in both conditions, we investigated differences in white matter neurite density depending on whether tract fibres were traversing white matter T_2_-hyperintense lesions. When studying unsegmented white matter tracts we have found a significant decrease in NDI, as compared to HC, in most white matter tracts in multiple sclerosis, but not in NMOSD. However, segmenting white matter tracts revealed a more distinctive pattern. In multiple sclerosis, a decrease in NDI was found in the vast majority of tract fibres, regardless of whether they were traversing (NDI decrease in 40/50 tracts) or not traversing (NDI decrease in 37/50 tracts) through white matter lesions. In multiple sclerosis, a reduction in NDI in tracts not traversing through white matter lesions appeared to be associated with measures of cortical damage, but not PRLs. Interestingly, in NMOSD, NDI reductions were only observed in tract fibres traversing through lesions, but never in lesion-free fibres.

MS and NMOSD are distinct chronic inflammatory diseases of the CNS that typically follow a relapsing-remitting course. The differential diagnosis between multiple sclerosis and NMOSD relies on clinical differences (predilection of NMOSD to the optic nerve and the spinal cord damage with typically highly disabling attacks) and routine imaging followed by antibody testing but can be challenging in patients with overlapping presentations, few brain lesions or atypical imaging appearances. Despite a clinical and radiological overlap, there are major pathological differences between multiple sclerosis and NMOSD, which can be explored by microstructural imaging. In particular, multiple sclerosis is characterized by diffuse neurodegeneration, which has been discussed to be caused by Wallerian degeneration of axons transected by remote lesions,^40^ smouldering inflammation associated with microglial activation,^41^ and cortical pathology.^42^ In NMOSD axonal loss is thought to be mainly secondary to inflammatory attacks, while the existence of attack-independent neurodegeneration remains controversial.^43^

Our results suggest that diffuse NAWM neurodegeneration in multiple sclerosis cannot be fully explained by Wallerian degeneration triggered by remote lesions as tract fibres which have not crossed any white matter lesions at any location typically had decreased NDI values when compared with corresponding tract fibres in cerebral white matter from HC. Interestingly, PRLs, which are suggestive of smouldering inflammation at the lesion edge, were not associated with lower NDI values in non-lesioned tracts. On the other hand, T_1_ relaxation rates for the cerebral cortex, identified as highly sensitive MRI measures of cortical demyelination in multiple sclerosis,^23^ were lowered in the majority (103/150) of cortical regions in our multiple sclerosis patients as compared to HC, a finding that was only present in 4 out of 150 cortical regions in NMOSD patients. In multiple sclerosis, we have also found a significant negative correlation between the number of cortical lesions and NDI in the majority of lesion-free white matter tracts, potentially suggesting the effect of cortical pathology on diffuse neurodegeneration in NAWM in multiple sclerosis. Interestingly, as compared to HC, the lowered T_1_ relaxation rates found in our NMOSD patients were located bilaterally in the central sulcus area associated with motor and sensory functions. We believe that this particular finding could reflect cortical changes in response to chronic nerve damage in the spinal cord as 18/20 NMOSD patients in our cohort had a history of transverse myelitis.

While non-conventional imaging has the potential to discriminate between NMOSD and multiple sclerosis, previous studies aiming at characterizing NAWM in NMOSD showed conflicting results. In this study in NMOSD, we have found significant NDI reductions in several tract fibres traversing through white matter lesions, but not in fibres that were anatomically independent of them. This finding suggests that in contrast to multiple sclerosis, neurodegeneration independent of lesions is largely absent in NMOSD. In particular, we have identified six tracts, where fibres not traversing through white matter lesions had significantly lower NDI in multiple sclerosis when compared with NMOSD. These tracts have been linked to pyramidal (left corticospinal tract), cerebellar (middle cerebellar peduncle, left inferior cerebellar peduncle, left parieto-occipital pontine tract) and cognitive (left inferior longitudinal fascicle, isthmus of the corpus callosum) functions. The cerebellar discriminator appears to be particularly interesting since previous studies showed that the loss of fractional anisotropy (FA) in the cerebellum without white matter lesions was already present in patients with early/mild multiple sclerosis and without any cerebellar volume reductions.^20,44^ Moreover, loss of FA in the cerebellum was also found in patients with clinically isolated syndrome who did not have any infratentorial lesions, and was associated with shorter conversion time to clinically definite multiple sclerosis.^45^ We have also identified one tract in which fibres traversing through white matter lesions (isthmus of the corpus callosum) had significantly lower NDI in multiple sclerosis than in NMOSD patients.

There are several strengths and limitations to our study. Due to the rarity of NMOSD, the results are drawn from a relatively small cohort of patients. Multiple sclerosis patients were on average younger and less disabled than NMOSD patients, which represented natural differences between the two conditions with NMOSD presenting at an older age and being a highly disabling condition early on.^1^ Despite these differences, multiple sclerosis was associated with greater white matter and cortical damage when compared with NMOSD and HC. This result combined with the findings from Cox *et al.*,^46^ who demonstrated NDI decrease from middle to old age might imply that the effect observed in the current study might be even greater on age-balanced groups. Tract segmentation into parts traversing and not traversing through white matter lesions was based on visible hyperintensities obtained from conventional FLAIR images and did not take into account lesions that might have resolved over time. It is, however, worth noting that in multiple sclerosis white matter lesions tend to accumulate in time rather than resolve.^47^ The strength of this study lies in its novel approach combining lesion-informed tractometry and optimized biologically plausible imaging measurements allowing for a detailed assessment of NAWM in well-defined disease cohorts studied in a prospective research MRI setting. To our knowledge, this is the first study in which the effect of disease processes in multiple sclerosis and NMOSD was assessed separately in white matter tracts segmented into fibres traversing and not traversing through white matter lesions. We argue that the segmentation along with the exclusion of lesioned white matter from the analysis has allowed us to show a more detailed picture of NAWM damage patterns that would otherwise be obscured by lesion load.

## Conclusion

We have identified, and we believe for the first time, specific non-conventional imaging differences between multiple sclerosis and AQP4 antibody NMOSD, which correspond to their distinct pathology in the cerebral white matter, and in the future can be tested as diagnostic discriminators in separate cohorts. In particular, a decrease in NODDI-derived NDI was a common finding in white matter tracts in multiple sclerosis, but was rarely seen in NMOSD and if present it was limited only to tracts traversing white matter lesions. NODDI measurements are feasible in clinical practice but would require using multi-shell diffusion-based protocols which are available in most modern clinical scanners. However, further reproducibility studies and establishing cut-offs for HC are needed.^21,48^ If validated on well-defined multiple sclerosis and AQP4 NMOSD cohorts, non-conventional MRI metrics could be implemented to help diagnose challenging patients with few brain lesions or borderline conventional MRI presentations.^49^

## Supplementary Material

fcae295_Supplementary_Data

## Data Availability

Data is available from the corresponding author upon request. Shell and Python scripts used in data pre-processing and analysis are located in the following GitHub repository https://github.com/nencki-lobi/NAWA/.
