# Supplementary material for: Patterns of cerebral damage in multiple sclerosis and aquaporin-4 antibody-positive neuromyelitis optica spectrum disorders—major differences revealed by non-conventional imaging
Source: Brain Commun. 2024 Aug 30;6(5):fcae295. doi: 10.1093/braincomms/fcae295 (PMC11384145; doi:10.1093/braincomms/fcae295)
Supplement: fcae295_Supplementary_Data [file fcae295_supplementary_data.pdf]

SUPPLEMENTARY MATERIALS

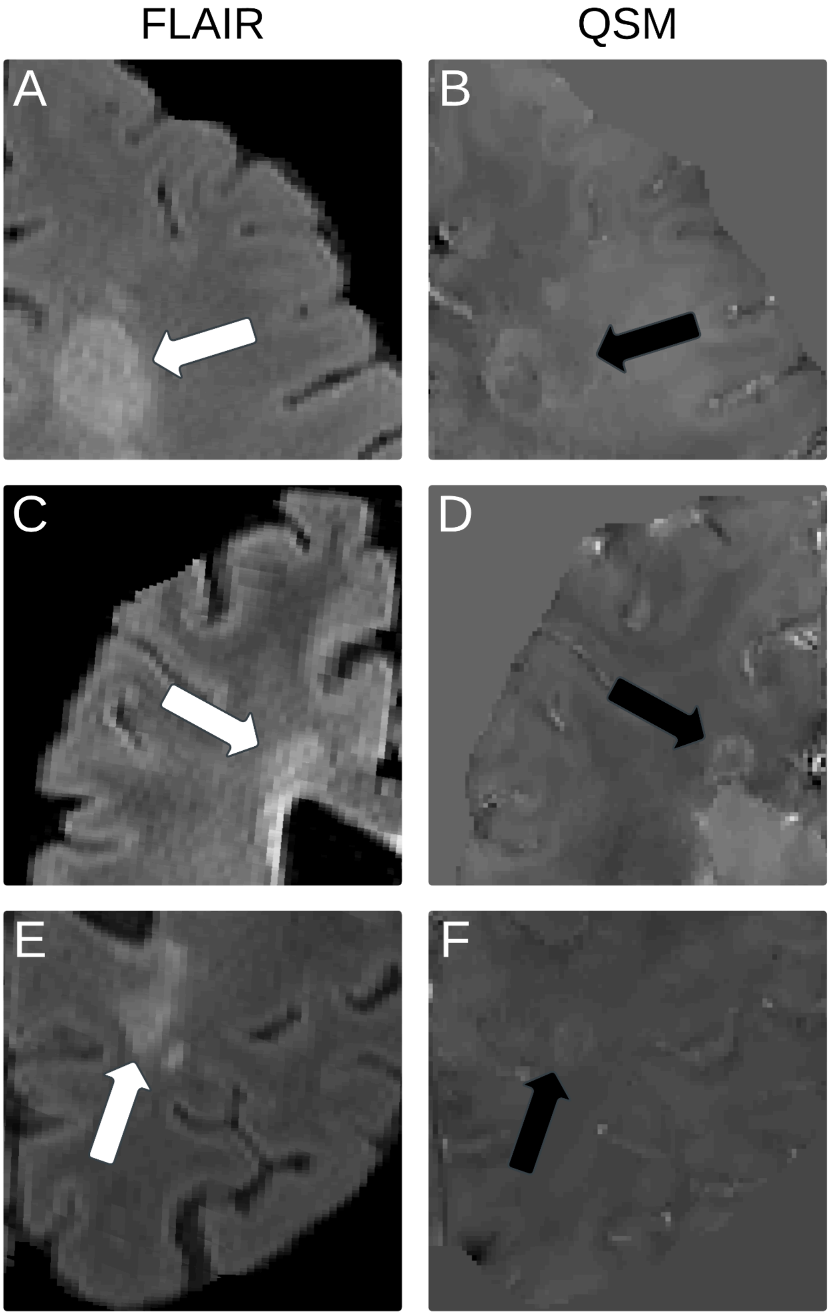

Supplementary Figure 1 Examples of paramagnetic rim lesions visible on FLAIR (depicted by white arrows) and corresponding QSM images (depicted by black arrows). (**A, B**) 29 year old male with RRMS. (**C, D**) 42 year old female with RRMS. (**E, F**) 45 year old female with RRMS.

QSM quantitative susceptibility mapping; FLAIR fluid attenuated inversion recovery; RRMS relapsing-remitting multiple sclerosis.

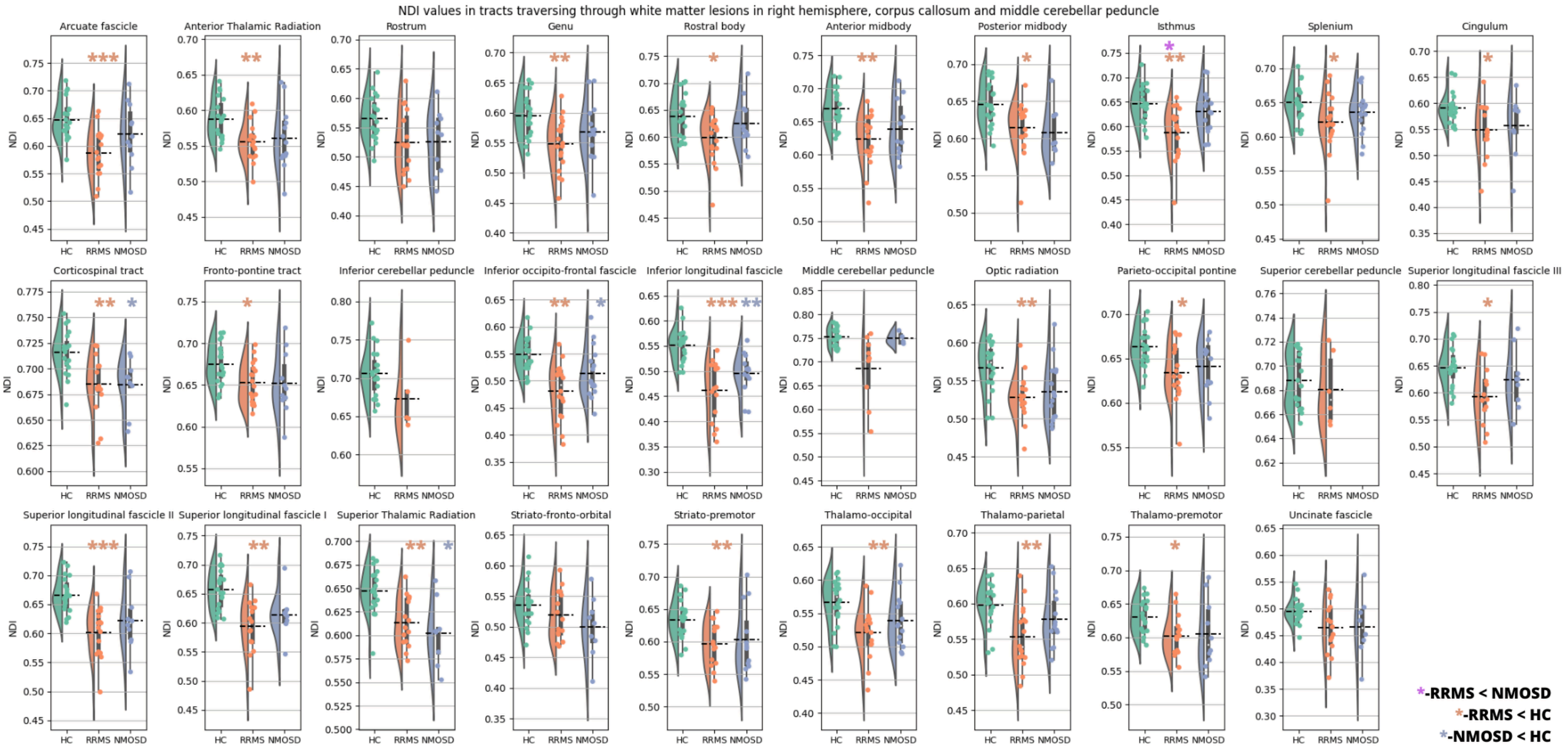

Supplementary Figure 2 NDI values in tract fibres traversing through white matter lesions in the right hemisphere, corpus callosum, and middle cerebellar peduncle. Significant results of mean NDI differences assessed with Welch's t-tests. Each data point in each tract represents a value from a single participant.

*p<0.05, **p < 0.01, ***p < 0.001; RRMS = relapsing-remitting multiple sclerosis; NMOSD = neuromyelitis optica spectrum disorders; HC = healthy controls

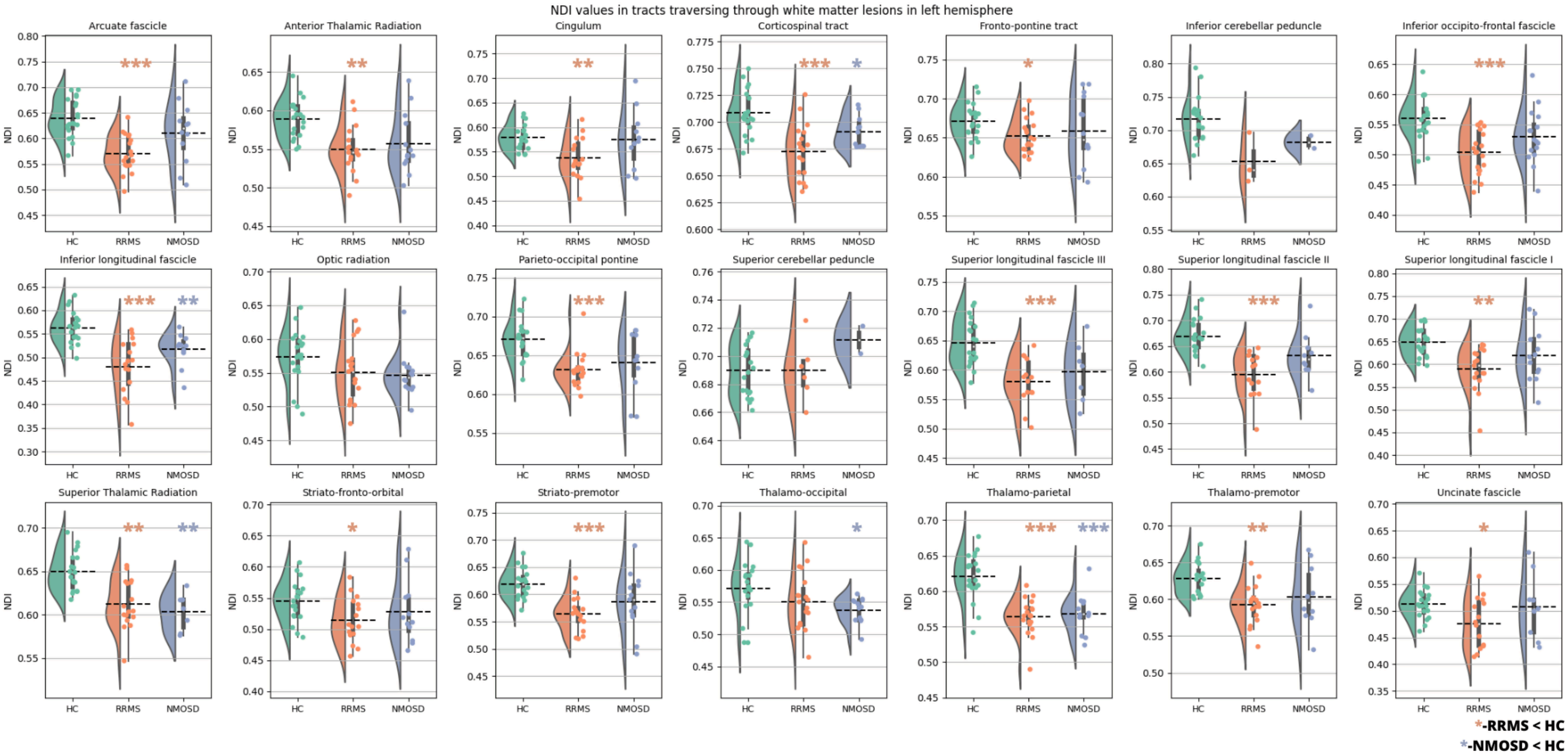

Supplementary Figure 3 NDI values in tract fibres traversing through white matter lesions in left hemisphere. Significant results of mean NDI differences assessed with Welch's t-tests. Each data point in each tract represents a value from a single participant.

*p<0.05, **p < 0.01, ***p < 0.001; RRMS = relapsing-remitting multiple sclerosis; NMOSD = neuromyelitis optica spectrum disorders; HC = healthy controls

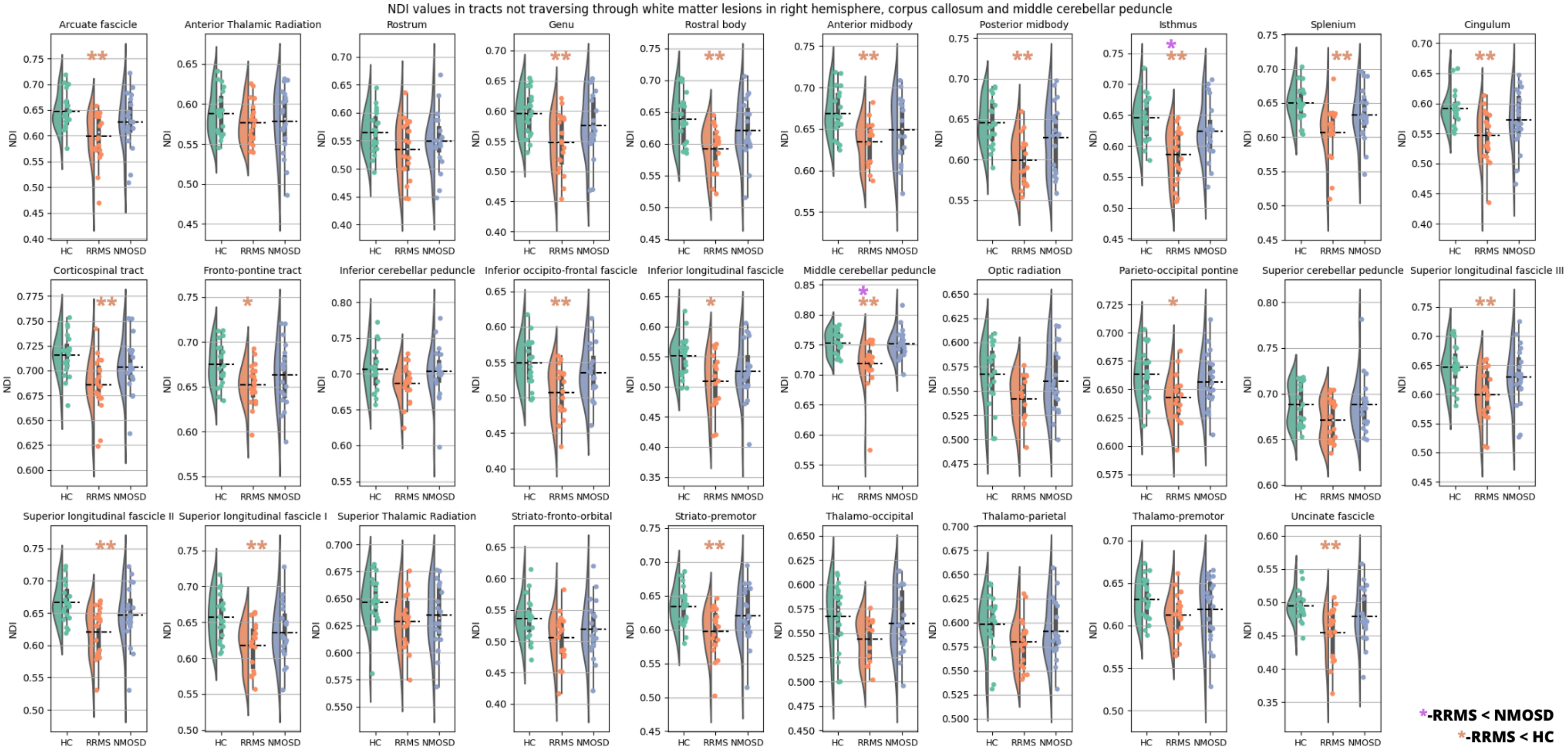

Supplementary Figure 4 NDI values in tract fibres not traversing through white matter lesions in the right hemisphere, corpus callosum, and middle cerebellar peduncle. Significant results of mean NDI differences assessed with Welch's t-tests. Each data point in each tract represents a value from a single participant.

*p<0.05, **p < 0.01, ***p < 0.001; RRMS = relapsing-remitting multiple sclerosis; NMOSD = neuromyelitis optica spectrum disorders; HC = healthy controls

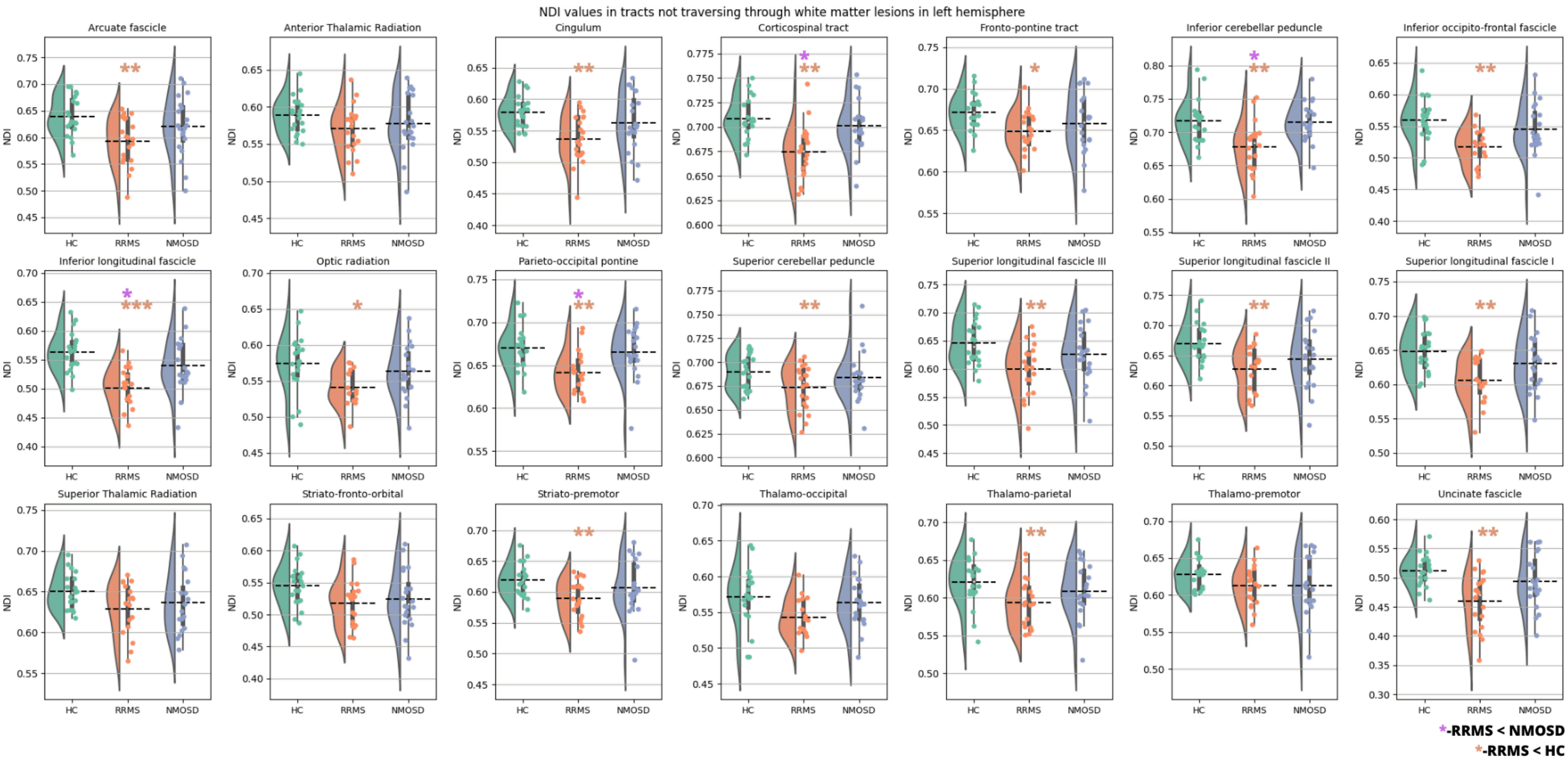

Supplementary Figure 5 NDI values in tract fibres not traversing through white matter lesions in the left hemisphere. Significant results of mean NDI differences assessed with Welch's t-tests. Each data point in each tract represents a value from a single participant.

*p<0.05, **p < 0.01, ***p < 0.001; RRMS = relapsing-remitting multiple sclerosis; NMOSD = neuromyelitis optica spectrum disorders; HC = healthy controls

Supplementary Table 1

Comparison of NDI between MS and NMOSD in unsegmented white matter tracts

Tract	MS		NMOSD		T-value	Cohen's d	p-value (FDR corrected)
	M	***SD***	***M***	***SD***			
AF_left	0,58	0,06	0,62	0,06	-2,12	-0,67	0,08
AF_right	0,59	0,05	0,63	0,05	-1,95	-0,62	0,10
ATR_left	0,56	0,04	0,57	0,04	-1,18	-0,37	0,27
ATR_right	0,56	0,03	0,57	0,04	-0,95	-0,30	0,37
CC_1	0,53	0,06	0,55	0,05	-1,03	-0,32	0,33
CC_2	0,54	0,06	0,57	0,05	-1,94	-0,61	0,10
CC_3	0,58	0,06	0,62	0,05	-2,24	-0,71	0,07
CC_4	0,61	0,05	0,64	0,05	-2,22	-0,71	0,07
CC_5	0,60	0,04	0,63	0,04	-2,46	-0,78	**0,04***
CC_6	0,57	0,06	0,62	0,05	-2,92	-0,92	**0,02***
CC_7	0,57	0,06	0,63	0,04	-3,31	-1,05	**0,01****
CG_left	0,53	0,05	0,56	0,05	-2,02	-0,64	0,09
CG_right	0,55	0,04	0,57	0,05	-1,82	-0,57	0,12
CST_left	0,67	0,03	0,70	0,03	-3,26	-1,03	**0,01****
CST_right	0,68	0,03	0,70	0,03	-1,90	-0,60	0,11
FPT_left	0,64	0,03	0,66	0,04	-1,14	-0,36	0,28
FPT_right	0,65	0,03	0,66	0,04	-1,48	-0,47	0,18
ICP_left	0,68	0,04	0,71	0,03	-3,51	-1,11	**0,01****
ICP_right	0,69	0,03	0,70	0,04	-1,78	-0,56	0,12
IFO_left	0,48	0,06	0,54	0,05	-3,30	-1,04	**0,01****
IFO_right	0,47	0,08	0,53	0,04	-2,81	-0,89	**0,02***
ILF_left	0,47	0,07	0,54	0,05	-3,74	-1,18	**0,01***
ILF_right	0,46	0,09	0,52	0,05	-2,67	-0,84	**0,03***
MCP	0,72	0,04	0,75	0,03	-3,12	-0,99	**0,01***
OR_left	0,50	0,06	0,55	0,04	-3,14	-0,99	**0,01***
OR_right	0,50	0,07	0,55	0,04	-2,64	-0,83	**0,03***
POPT_left	0,62	0,04	0,66	0,03	-3,13	-0,99	**0,01***
POPT_right	0,62	0,04	0,65	0,03	-2,92	-0,92	**0,02***
SCP_left	0,67	0,02	0,68	0,03	-1,31	-0,41	0,22
SCP_right	0,67	0,02	0,69	0,03	-1,91	-0,60	0,11
SLF_III_left	0,60	0,05	0,63	0,05	-1,87	-0,59	0,11
SLF_III_right	0,60	0,05	0,63	0,05	-2,10	-0,66	0,08
SLF_II_left	0,61	0,06	0,64	0,05	-1,69	-0,53	0,14
SLF_II_right	0,61	0,05	0,64	0,05	-1,92	-0,61	0,11
SLF_I_left	0,60	0,05	0,63	0,04	-2,19	-0,69	0,07
SLF_I_right	0,61	0,05	0,63	0,04	-1,90	-0,60	0,11

STR_left	0,61	0,04	0,63	0,04	-2,03	-0,65	0,09
STR_right	0,61	0,04	0,63	0,04	-1,46	-0,46	0,19
ST_FO_left	0,51	0,04	0,52	0,05	-0,92	-0,29	0,38
ST_FO_right	0,50	0,05	0,52	0,05	-1,19	-0,38	0,27
ST_PREM_left	0,58	0,04	0,60	0,05	-1,61	-0,51	0,16
ST_PREM_right	0,59	0,04	0,62	0,04	-1,87	-0,59	0,11
T_OCC_left	0,50	0,07	0,55	0,04	-3,21	-1,01	**0,01***
T_OCC_right	0,49	0,07	0,55	0,04	-2,72	-0,86	**0,03***
T_PAR_left	0,56	0,05	0,59	0,04	-2,00	-0,63	0,10
T_PAR_right	0,55	0,05	0,58	0,04	-2,76	-0,87	**0,02***
T_PREM_left	0,60	0,03	0,61	0,04	-0,68	-0,21	0,51
T_PREM_right	0,61	0,03	0,62	0,04	-0,94	-0,30	0,37
UF_left	0,46	0,05	0,49	0,05	-2,25	-0,71	0,07
UF_right	0,45	0,04	0,48	0,04	-2,03	-0,64	0,09

Comparison of NDI between MS and NMOSD in white matter fibers traversing through white matter lesions

Tract	MS		NMOSD		T-value	Cohen's d	p-value (FDR corrected)
	M	***SD***	***M***	***SD***			
AF_right	0,59	0,04	0,62	0,06	-1,69	-0,69	0,18
ATR_left	0,55	0,03	0,56	0,04	-0,59	-0,22	0,64
ATR_right	0,56	0,03	0,56	0,04	-0,34	-0,13	0,78
CC_1	0,53	0,05	0,53	0,05	-0,06	-0,02	0,96
CC_2	0,55	0,04	0,57	0,05	-1,17	-0,42	0,34
CC_3	0,60	0,04	0,63	0,04	-1,63	-0,60	0,19
CC_4	0,62	0,04	0,64	0,04	-0,93	-0,36	0,45
CC_5	0,62	0,03	0,61	0,04	0,46	0,20	0,72
CC_6	0,59	0,05	0,63	0,05	-2,56	-0,87	**0,04***
CC_7	0,62	0,04	0,64	0,03	-1,16	-0,39	0,34
CG_left	0,54	0,04	0,58	0,06	-0,99	-0,30	0,16
CG_right	0,55	0,05	0,56	0,06	-0,35	-0,15	0,78
CST_left	0,67	0,03	0,69	0,02	-2,44	-0,82	0,06
CST_right	0,69	0,03	0,69	0,03	0,06	0,02	0,96
FPT_left	0,65	0,02	0,66	0,04	-0,50	-0,20	0,69
FPT_right	0,65	0,02	0,65	0,04	0,06	0,02	0,96
ICP_left	0,65	0,04	0,68	0,01	-1,20	-0,89	0,41
IFO_left	0,50	0,04	0,53	0,05	-1,85	-0,64	0,14
IFO_right	0,48	0,05	0,51	0,05	-1,96	-0,66	0,12
ILF_left	0,48	0,06	0,52	0,04	-2,01	-0,72	0,11
ILF_right	0,46	0,06	0,50	0,04	-1,79	-0,64	0,15
MCP	0,69	0,07	0,75	0,01	-2,60	-1,04	0,07
OR_left	0,55	0,04	0,55	0,03	0,33	0,11	0,79
OR_right	0,53	0,03	0,54	0,04	-0,60	-0,22	0,63

POPT_left	0,63	0,02	0,64	0,04	-0,70	-0,31	0,57
POPT_right	0,63	0,03	0,64	0,03	-0,61	-0,23	0,63
SCP_left	0,69	0,02	0,71	0,01	-1,65	-1,05	0,28
SLF_III_left	0,58	0,04	0,60	0,05	-0,76	-0,38	0,55
SLF_III_right	0,59	0,05	0,62	0,06	-1,34	-0,59	0,28
SLF_II_left	0,60	0,04	0,63	0,04	-2,03	-0,83	0,12
SLF_II_right	0,60	0,04	0,62	0,05	-1,05	-0,44	0,39
SLF_I_left	0,59	0,05	0,62	0,06	-1,49	-0,56	0,23
SLF_I_right	0,59	0,05	0,61	0,04	-1,08	-0,46	0,39
STR_left	0,61	0,03	0,60	0,02	0,84	0,34	0,50
STR_right	0,61	0,03	0,60	0,03	0,82	0,37	0,51
ST_FO_left	0,51	0,04	0,53	0,05	-0,79	-0,32	0,52
ST_FO_right	0,52	0,04	0,50	0,05	1,00	0,44	0,42
ST_PREM_left	0,56	0,03	0,59	0,05	-1,38	-0,55	0,27
ST_PREM_right	0,60	0,03	0,60	0,05	-0,40	-0,16	0,75
T_OCC_left	0,55	0,04	0,54	0,02	1,13	0,36	0,35
T_OCC_right	0,52	0,04	0,54	0,04	-1,23	-0,45	0,32
T_PAR_left	0,56	0,03	0,57	0,03	-0,48	-0,18	0,70
T_PAR_right	0,55	0,04	0,58	0,04	-1,71	-0,61	0,17
T_PREM_left	0,59	0,03	0,60	0,04	-0,71	-0,32	0,57
T_PREM_right	0,60	0,03	0,61	0,05	-0,19	-0,09	0,88
UF_left	0,48	0,05	0,51	0,06	-1,34	-0,60	0,28
UF_right	0,46	0,05	0,47	0,06	-0,07	-0,03	0,96

Comparison of NDI between MS and NMOSD in white matter fibers not traversing through white matter lesions

Tract	MS		NMOSD		T-value	Cohen's d	p-value (FDR corrected)
	M	***SD***	***M***	***SD***			
AF_left	0,59	0,05	0,62	0,06	-1,73	-0,55	0,17
AF_right	0,60	0,05	0,63	0,05	-1,74	-0,55	0,17
ATR_left	0,57	0,03	0,58	0,04	-0,64	-0,20	0,56
ATR_right	0,58	0,03	0,58	0,04	-0,20	-0,06	0,85
CC_1	0,54	0,05	0,55	0,05	-0,92	-0,29	0,42
CC_2	0,55	0,05	0,58	0,05	-1,76	-0,56	0,17
CC_3	0,59	0,04	0,62	0,05	-2,09	-0,68	0,11
CC_4	0,63	0,03	0,65	0,04	-1,28	-0,43	0,28
CC_5	0,60	0,03	0,63	0,04	-2,33	-0,74	0,08
CC_6	0,59	0,04	0,62	0,05	-2,70	-0,85	**0,04***
CC_7	0,61	0,04	0,63	0,04	-1,99	-0,65	0,13
CG_left	0,54	0,04	0,56	0,05	-1,92	-0,61	0,13
CG_right	0,55	0,04	0,57	0,05	-1,82	-0,57	0,15
CST_left	0,68	0,03	0,70	0,03	-3,08	-0,97	**0,02***
CST_right	0,69	0,03	0,70	0,03	-2,06	-0,65	0,11

FPT_left	0,65	0,02	0,66	0,04	-0,96	-0,30	0,40
FPT_right	0,65	0,02	0,66	0,04	-1,19	-0,38	0,30
ICP_left	0,68	0,04	0,72	0,03	-3,40	-1,08	**0,01****
ICP_right	0,69	0,03	0,70	0,04	-1,62	-0,51	0,19
IFO_left	0,52	0,03	0,55	0,04	-2,41	-0,77	0,07
IFO_right	0,51	0,04	0,54	0,04	-2,29	-0,74	0,08
ILF_left	0,50	0,03	0,54	0,05	-2,99	-0,95	**0,02***
ILF_right	0,51	0,05	0,53	0,05	-1,04	-0,34	0,37
MCP	0,72	0,04	0,75	0,03	-3,11	-0,98	**0,02***
OR_left	0,54	0,03	0,56	0,04	-2,10	-0,68	0,11
OR_right	0,54	0,03	0,56	0,03	-1,75	-0,59	0,17
POPT_left	0,64	0,02	0,67	0,03	-2,73	-0,86	**0,04***
POPT_right	0,64	0,02	0,66	0,02	-1,93	-0,62	0,13
SCP_left	0,67	0,02	0,68	0,03	-1,33	-0,42	0,27
SCP_right	0,67	0,02	0,69	0,03	-1,93	-0,61	0,13
SLF_III_left	0,60	0,05	0,63	0,05	-1,75	-0,55	0,17
SLF_III_right	0,60	0,05	0,63	0,05	-1,97	-0,62	0,13
SLF_II_left	0,63	0,04	0,64	0,05	-1,21	-0,38	0,30
SLF_II_right	0,62	0,04	0,65	0,05	-2,00	-0,63	0,12
SLF_I_left	0,61	0,03	0,63	0,04	-2,07	-0,66	0,11
SLF_I_right	0,62	0,03	0,64	0,04	-1,53	-0,49	0,22
STR_left	0,63	0,03	0,64	0,04	-0,65	-0,21	0,56
STR_right	0,63	0,03	0,64	0,03	-0,61	-0,20	0,58
ST_FO_left	0,52	0,04	0,52	0,05	-0,47	-0,15	0,67
ST_FO_right	0,51	0,04	0,52	0,05	-0,97	-0,31	0,40
ST_PREM_left	0,59	0,03	0,61	0,04	-1,51	-0,48	0,22
ST_PREM_right	0,60	0,04	0,62	0,04	-1,88	-0,59	0,14
T_OCC_left	0,54	0,03	0,56	0,04	-1,91	-0,62	0,13
T_OCC_right	0,54	0,02	0,56	0,03	-1,67	-0,56	0,19
T_PAR_left	0,59	0,03	0,61	0,04	-1,40	-0,44	0,24
T_PAR_right	0,58	0,03	0,59	0,03	-1,25	-0,40	0,29
T_PREM_left	0,61	0,03	0,61	0,04	-0,07	-0,02	0,95
T_PREM_right	0,61	0,03	0,62	0,04	-0,67	-0,21	0,55
UF_left	0,46	0,05	0,49	0,05	-2,31	-0,73	0,08
UF_right	0,46	0,04	0,48	0,04	-1,94	-0,61	0,13

*p < .05, **p < 0.01; NMOSD = neuromyelitis optica spectrum disorders; MS = multiple sclerosis; NDI = neurite density index; AF = arcuate fascicle; ATR = anterior thalamic radiation; CC = corpus callosum; CC_1 = rostrum; CC_2 = genu; CC_3 = rostral body; CC_4 = anterior midbody; CC_5 = posterior midbody; CC_6 = isthmus; CC_7 = splenium; CG = cingulum; CST = corticospinal tract; FPT = fronto-pontine tract; ICP = inferior cerebellar peduncle; IFO = inferior occipito-frontal fascicle; ILF = inferior longitudinal fascicle; MCP = middle cerebellar peduncle; OR = optic radiation; POPT = parieto-occipital pontine tract; SCP = superior cerebellar peduncle; SLF_I = superior longitudinal fascicle I; SLF_II = superior longitudinal fascicle II; SLF_III = superior longitudinal fascicle III; STR = superior thalamic radiation; ST_FO = striato-fronto-orbital; ST_PREM = striato-premotor; T_OCC = thalamo-occipital; T_PAR = thalamo-parietal; T_PREM = thalamo-premotor; UF = uncinate fascicle,

Supplementary Table 2

Comparison of NDI in unsegmented white matter tracts between in MS and whole tracts in HC

Tract	MS		HC		T-value	Cohen's d	p-value (FDR corrected)
	M	*SD*	*M*	*SD*			
AF_left	0,58	0,06	0,64	0,04	-3,71	-1,17	**0,00****
AF_right	0,59	0,05	0,65	0,04	-3,71	-1,17	**0,00****
ATR_left	0,56	0,04	0,59	0,02	-3,01	-0,95	**0,01***
ATR_right	0,56	0,03	0,59	0,03	-2,77	-0,88	**0,02***
CC_1	0,53	0,06	0,56	0,04	-2,28	-0,72	0,06
CC_2	0,54	0,06	0,60	0,04	-3,84	-1,22	**0,00****
CC_3	0,58	0,06	0,64	0,04	-3,98	-1,26	**0,00****
CC_4	0,61	0,05	0,67	0,03	-4,74	-1,50	**0,00****
CC_5	0,59	0,04	0,65	0,03	-4,51	-1,43	**0,00****
CC_6	0,57	0,06	0,65	0,04	-4,66	-1,47	**0,00****
CC_7	0,57	0,06	0,65	0,03	-5,04	-1,59	**0,00****
CG_left	0,53	0,05	0,58	0,03	-3,91	-1,24	**0,00****
CG_right	0,55	0,04	0,59	0,03	-3,78	-1,19	**0,00****
CST_left	0,67	0,03	0,71	0,02	-4,67	-1,48	**0,00****
CST_right	0,68	0,03	0,72	0,02	-3,84	-1,21	**0,00****
FPT_left	0,64	0,03	0,67	0,02	-3,51	-1,11	**0,00****
FPT_right	0,65	0,03	0,68	0,02	-3,35	-1,06	**0,01***
ICP_left	0,68	0,04	0,72	0,03	-3,59	-1,14	**0,00****
ICP_right	0,69	0,02	0,71	0,03	-2,42	-0,77	**0,05***
IFO_left	0,48	0,06	0,56	0,03	-4,96	-1,57	**0,00****
IFO_right	0,47	0,08	0,55	0,03	-4,23	-1,34	**0,00****
ILF_left	0,47	0,07	0,56	0,03	-5,38	-1,70	**0,00****
ILF_right	0,46	0,09	0,55	0,03	-4,38	-1,38	**0,00****
MCP	0,72	0,04	0,75	0,02	-3,44	-1,09	0,01
OR_left	0,50	0,06	0,57	0,04	-4,50	-1,42	**0,00****
OR_right	0,50	0,07	0,57	0,03	-4,09	-1,29	**0,00****
POPT_left	0,62	0,04	0,67	0,03	-4,82	-1,52	**0,00****
POPT_right	0,62	0,04	0,66	0,02	-4,24	-1,34	**0,00****
SCP_left	0,67	0,02	0,69	0,02	-2,42	-0,76	**0,05***
SCP_right	0,67	0,02	0,69	0,02	-2,30	-0,73	0,06
SLF_III_left	0,59	0,05	0,65	0,04	-3,49	-1,11	**0,00****
SLF_III_right	0,60	0,05	0,65	0,04	-3,72	-1,18	**0,00****
SLF_II_left	0,61	0,06	0,67	0,03	-3,76	-1,19	**0,00****
SLF_II_right	0,61	0,05	0,67	0,03	-4,34	-1,37	**0,00****
SLF_I_left	0,60	0,05	0,65	0,03	-4,13	-1,31	**0,00****
SLF_I_right	0,61	0,05	0,66	0,03	-3,93	-1,24	**0,00****

STR_left	0,61	0,04	0,65	0,02	-4,46	-1,41	**0,00****
STR_right	0,61	0,04	0,65	0,02	-3,65	-1,15	**0,00****
ST_FO_left	0,51	0,04	0,54	0,03	-3,10	-0,98	**0,01***
ST_FO_right	0,50	0,05	0,54	0,03	-2,97	-0,94	**0,02***
ST_PREM_left	0,58	0,04	0,62	0,03	-3,77	-1,19	**0,00****
ST_PREM_right	0,59	0,04	0,63	0,03	-3,76	-1,19	**0,00****
T_OCC_left	0,50	0,07	0,57	0,04	-4,27	-1,35	**0,00****
T_OCC_right	0,49	0,07	0,57	0,03	-4,01	-1,27	**0,00****
T_PAR_left	0,56	0,05	0,62	0,03	-4,16	-1,32	**0,00****
T_PAR_right	0,55	0,05	0,60	0,03	-4,19	-1,33	**0,00****
T_PREM_left	0,60	0,03	0,63	0,02	-2,84	-0,90	**0,02***
T_PREM_right	0,61	0,03	0,63	0,02	-2,80	-0,89	**0,02***
UF_left	0,46	0,05	0,51	0,03	-4,36	-1,38	**0,00****
UF_right	0,45	0,04	0,49	0,02	-3,75	-1,19	**0,00****

Comparison of NDI in white matter fibers traversing through white matter lesions in MS and whole tracts in HC

Tract	MS		HC		T-value	Cohen's d	p-value (FDR corrected)
	M	***SD***	***M***	***SD***			
AF_left	0,57	0,04	0,64	0,04	-5,75	-1,87	**0,00*****
AF_right	0,59	0,04	0,65	0,04	-4,41	-1,51	**0,01****
ATR_left	0,55	0,03	0,59	0,02	-4,28	-1,44	**0,01****
ATR_right	0,56	0,03	0,59	0,03	-3,47	-1,14	**0,01****
CC_1	0,52	0,05	0,56	0,04	-2,53	-0,88	0,05
CC_2	0,55	0,04	0,60	0,04	-3,69	-1,19	**0,01****
CC_3	0,60	0,04	0,64	0,04	-3,01	-0,97	**0,02***
CC_4	0,62	0,04	0,67	0,03	-4,15	-1,34	**0,01****
CC_5	0,61	0,03	0,65	0,03	-2,95	-0,97	**0,02***
CC_6	0,59	0,05	0,65	0,04	-4,01	-1,30	**0,01****
CC_7	0,62	0,04	0,65	0,03	-2,56	-0,83	0,05
CG_left	0,54	0,04	0,58	0,03	-3,58	-1,27	**0,01****
CG_right	0,55	0,05	0,59	0,03	-2,82	-1,04	**0,03***
CST_left	0,67	0,03	0,71	0,02	-4,70	-1,55	**0,00*****
CST_right	0,69	0,03	0,72	0,02	-3,63	-1,25	**0,01****
FPT_left	0,65	0,02	0,67	0,02	-2,76	-0,88	**0,03***
FPT_right	0,65	0,02	0,68	0,02	-2,86	-0,93	**0,02***
ICP_left	0,65	0,04	0,72	0,03	-2,72	-1,92	0,16
ICP_right	0,67	0,05	0,71	0,03	-1,55	-0,99	0,27
IFO_left	0,50	0,04	0,56	0,03	-4,88	-1,56	**0,00*****
IFO_right	0,48	0,05	0,55	0,03	-4,78	-1,59	**0,00*****
ILF_left	0,48	0,06	0,56	0,03	-5,05	-1,79	**0,00*****
ILF_right	0,46	0,06	0,55	0,03	-5,46	-1,94	**0,00*****

MCP	0,69	0,07	0,75	0,02	-2,77	-1,59	0,06
OR_left	0,55	0,04	0,57	0,04	-1,72	-0,55	0,16
OR_right	0,53	0,03	0,57	0,03	-3,65	-1,22	0,00
POPT_left	0,63	0,02	0,67	0,03	-5,16	-1,65	**0,00*** **
POPT_right	0,63	0,03	0,66	0,02	-3,23	-1,07	**0,01***
SCP_left	0,69	0,02	0,69	0,02	-0,01	0,00	0,99
SCP_right	0,68	0,03	0,69	0,02	-0,55	-0,30	0,67
SLF_III_left	0,58	0,04	0,65	0,04	-4,77	-1,67	**0,00*** **
SLF_III_right	0,59	0,05	0,65	0,04	-3,28	-1,26	0,02
SLF_II_left	0,60	0,04	0,67	0,03	-5,55	-1,98	**0,00*** **
SLF_II_right	0,60	0,04	0,67	0,03	-5,12	-1,79	**0,00*** **
SLF_I_left	0,59	0,05	0,65	0,03	-4,37	-1,49	**0,01****
SLF_I_right	0,59	0,05	0,66	0,03	-4,56	-1,63	**0,01****
STR_left	0,61	0,03	0,65	0,02	-4,43	-1,50	**0,01****
STR_right	0,61	0,03	0,65	0,02	-3,89	-1,35	**0,01****
ST_FO_left	0,51	0,04	0,54	0,03	-2,66	-0,90	**0,04***
ST_FO_right	0,52	0,04	0,54	0,03	-1,32	-0,46	0,28
ST_PREM_left	0,56	0,03	0,62	0,03	-5,98	-1,95	**0,00*** **
ST_PREM_right	0,60	0,03	0,63	0,03	-3,48	-1,21	**0,01****
T_OCC_left	0,55	0,04	0,57	0,04	-1,53	-0,49	0,21
T_OCC_right	0,52	0,04	0,57	0,03	-3,68	-1,26	0,00
T_PAR_left	0,56	0,03	0,62	0,03	-6,14	-1,94	**0,00*** **
T_PAR_right	0,55	0,04	0,60	0,03	-3,68	-1,25	**0,01****
T_PREM_left	0,59	0,03	0,63	0,02	-4,33	-1,46	**0,01****
T_PREM_right	0,60	0,03	0,63	0,02	-2,95	-1,04	**0,02***
UF_left	0,48	0,05	0,51	0,03	-2,75	-0,98	**0,04***
UF_right	0,46	0,05	0,49	0,02	-2,28	-0,85	0,08

Comparison of NDI in white matter fibers not traversing through white matter lesions in MS and whole tracts in HC

Tract	MS		HC		T-value	Cohen's d	p-value (FDR corrected)
	M	*SD*	*M*	*SD*			
AF_left	0,59	0,05	0,64	0,04	-3,62	-1,14	**0,01****
AF_right	0,60	0,05	0,65	0,04	-3,52	-1,11	**0,01****
ATR_left	0,57	0,03	0,59	0,02	-2,05	-0,65	0,11
ATR_right	0,58	0,03	0,59	0,03	-1,34	-0,42	0,26
CC_1	0,53	0,05	0,56	0,04	-2,11	-0,67	0,11
CC_2	0,55	0,05	0,60	0,04	-3,59	-1,14	**0,01****
CC_3	0,59	0,04	0,64	0,04	-3,76	-1,22	**0,01****
CC_4	0,63	0,03	0,67	0,03	-3,67	-1,25	**0,01****
CC_5	0,60	0,03	0,65	0,03	-4,73	-1,54	**0,01****
CC_6	0,59	0,04	0,65	0,04	-4,75	-1,50	**0,01****

CC_7	0,61	0,04	0,65	0,03	-3,75	-1,24	**0,01****
CG_left	0,54	0,04	0,58	0,03	-4,19	-1,33	**0,01****
CG_right	0,55	0,04	0,59	0,03	-3,86	-1,22	**0,01****
CST_left	0,67	0,03	0,71	0,02	-4,41	-1,40	**0,01****
CST_right	0,69	0,03	0,72	0,02	-3,80	-1,20	**0,01****
FPT_left	0,65	0,02	0,67	0,02	-3,12	-0,99	**0,02***
FPT_right	0,65	0,02	0,68	0,02	-3,08	-0,97	**0,02***
ICP_left	0,68	0,04	0,72	0,03	-3,48	-1,10	**0,01****
ICP_right	0,69	0,03	0,71	0,03	-2,23	-0,71	0,09
IFO_left	0,52	0,03	0,56	0,03	-4,13	-1,34	**0,01****
IFO_right	0,51	0,04	0,55	0,03	-3,67	-1,20	**0,01****
ILF_left	0,50	0,03	0,56	0,03	-5,64	-1,81	**0,00*****
ILF_right	0,51	0,05	0,55	0,03	-3,06	-1,04	**0,02***
MCP	0,72	0,04	0,75	0,02	-3,46	-1,09	**0,01****
OR_left	0,54	0,03	0,57	0,04	-2,95	-0,95	**0,02***
OR_right	0,54	0,02	0,57	0,03	-2,54	-0,84	0,06
POPT_left	0,64	0,02	0,67	0,03	-3,69	-1,17	**0,01****
POPT_right	0,64	0,02	0,66	0,02	-2,94	-0,94	**0,02***
SCP_left	0,67	0,02	0,69	0,02	-2,46	-0,78	0,06
SCP_right	0,67	0,02	0,69	0,02	-2,29	-0,72	0,08
SLF_III_left	0,60	0,05	0,65	0,04	-3,44	-1,09	**0,01****
SLF_III_right	0,60	0,05	0,65	0,04	-3,63	-1,15	**0,01****
SLF_II_left	0,63	0,04	0,67	0,03	-3,74	-1,20	**0,01****
SLF_II_right	0,62	0,04	0,67	0,03	-4,25	-1,35	**0,01****
SLF_I_left	0,61	0,03	0,65	0,03	-4,21	-1,35	**0,01****
SLF_I_right	0,62	0,03	0,66	0,03	-3,77	-1,21	**0,01****
STR_left	0,63	0,03	0,65	0,02	-2,31	-0,77	0,08
STR_right	0,63	0,03	0,65	0,02	-2,20	-0,71	0,09
ST_FO_left	0,52	0,03	0,54	0,03	-2,52	-0,80	0,06
ST_FO_right	0,51	0,04	0,54	0,03	-2,59	-0,82	0,05
ST_PREM_left	0,59	0,03	0,62	0,03	-3,34	-1,06	**0,01****
ST_PREM_right	0,60	0,04	0,63	0,03	-3,59	-1,13	**0,01****
T_OCC_left	0,54	0,03	0,57	0,04	-2,43	-0,78	0,07
T_OCC_right	0,54	0,02	0,57	0,03	-2,37	-0,77	0,08
T_PAR_left	0,59	0,03	0,62	0,03	-2,62	-0,83	**0,05***
T_PAR_right	0,58	0,03	0,60	0,03	-2,07	-0,66	0,11
T_PREM_left	0,61	0,03	0,63	0,02	-2,08	-0,66	0,11
T_PREM_right	0,61	0,02	0,63	0,02	-2,33	-0,74	0,08
UF_left	0,46	0,05	0,51	0,03	-4,37	-1,38	**0,01****
UF_right	0,45	0,04	0,49	0,02	-3,93	-1,24	**0,01****

*p < ,05, **p < 0,01, p*** < ,001; MS = multiple sclerosis; HC = healthy controls; NDI = neurite density index; AF = arcuate fascicle; ATR = anterior thalamic radiation; CC = corpus callosum; CC_1 = rostrum; CC_2 = genu; CC_3 = rostral body; CC_4 = anterior midbody; CC_5 = posterior midbody; CC_6 = isthmus; CC_7 = splenium; CG = cingulum; CST = corticospinal tract; FPT = fronto-pontine tract; ICP = inferior cerebellar peduncle; IFO = inferior occipito-frontal fascicle; ILF = inferior longitudinal fascicle; MCP = middle cerebellar peduncle; OR = optic radiation; POPT = parieto-occipital pontine tract; SCP = superior cerebellar peduncle; SLF_I = superior longitudinal fascicle I; SLF_II = superior longitudinal fascicle II; SLF_III = superior longitudinal fascicle III; STR = superior thalamic radiation; ST_FO = striato-fronto-orbital; ST_PREM = striato-premotor; T_OCC = thalamo-occipital; T_PAR = thalamo-parietal; T_PREM = thalamo-premotor; UF = uncinate fascicle,

Supplementary Table 3

Comparison of NDI in unsegmented white matter tracts between NMOSD and HC

Tract	NMOSD		HC		T-value	Cohen's d	p-value (FDR corrected)
	M	*SD*	*M*	*SD*			
AF_left	0,62	0,06	0,64	0,04	1,48	0,47	0,18
AF_right	0,63	0,05	0,65	0,04	1,48	0,47	0,18
ATR_left	0,57	0,04	0,59	0,02	1,45	0,46	0,19
ATR_right	0,57	0,04	0,59	0,03	1,24	0,39	0,25
CC_1	0,55	0,05	0,56	0,04	1,16	0,37	0,27
CC_2	0,57	0,05	0,60	0,04	1,64	0,52	0,15
CC_3	0,62	0,05	0,64	0,04	1,60	0,51	0,16
CC_4	0,64	0,05	0,67	0,03	2,26	0,73	0,07
CC_5	0,63	0,04	0,65	0,03	1,62	0,51	0,16
CC_6	0,62	0,05	0,65	0,04	1,77	0,56	0,12
CC_7	0,63	0,04	0,65	0,03	2,14	0,68	0,08
CG_left	0,56	0,05	0,58	0,03	1,42	0,45	0,19
CG_right	0,57	0,05	0,59	0,03	1,43	0,45	0,19
CST_left	0,70	0,03	0,71	0,02	1,22	0,39	0,25
CST_right	0,70	0,03	0,72	0,02	1,84	0,58	0,11
FPT_left	0,66	0,04	0,67	0,02	1,55	0,49	0,17
FPT_right	0,66	0,04	0,68	0,02	1,35	0,43	0,21
ICP_left	0,71	0,03	0,72	0,03	0,23	0,07	0,82
ICP_right	0,70	0,04	0,71	0,03	0,32	0,10	0,76
IFO_left	0,54	0,05	0,56	0,03	1,73	0,55	0,13
IFO_right	0,53	0,04	0,55	0,03	1,86	0,59	0,11
ILF_left	0,54	0,05	0,56	0,03	1,87	0,59	0,11
ILF_right	0,52	0,05	0,55	0,03	2,24	0,71	0,07
MCP	0,75	0,02	0,75	0,02	0,24	0,08	0,82
OR_left	0,55	0,04	0,57	0,04	1,70	0,54	0,14
OR_right	0,54	0,04	0,57	0,03	1,84	0,58	0,11
POPT_left	0,66	0,03	0,67	0,03	1,56	0,49	0,17
POPT_right	0,65	0,03	0,66	0,02	1,39	0,44	0,20
SCP_left	0,68	0,03	0,69	0,02	0,84	0,26	0,42
SCP_right	0,69	0,03	0,69	0,02	0,02	0,01	0,98
SLF_III_left	0,63	0,05	0,65	0,04	1,44	0,45	0,19
SLF_III_right	0,63	0,05	0,65	0,04	1,29	0,41	0,23
SLF_II_left	0,64	0,05	0,67	0,03	2,13	0,67	0,08
SLF_II_right	0,64	0,05	0,67	0,03	1,91	0,60	0,11
SLF_I_left	0,63	0,04	0,65	0,03	1,75	0,55	0,13
SLF_I_right	0,63	0,04	0,66	0,03	2,02	0,64	0,09

STR_left	0,63	0,04	0,65	0,02	1,80	0,58	0,12
STR_right	0,63	0,03	0,65	0,02	1,96	0,62	0,10
ST_FO_left	0,52	0,05	0,54	0,03	1,92	0,61	0,11
ST_FO_right	0,52	0,05	0,54	0,03	1,51	0,48	0,18
ST_PREM_left	0,60	0,05	0,62	0,03	1,44	0,46	0,19
ST_PREM_right	0,62	0,04	0,63	0,03	1,48	0,47	0,18
T_OCC_left	0,55	0,04	0,57	0,04	1,50	0,48	0,18
T_OCC_right	0,55	0,04	0,57	0,03	1,77	0,56	0,12
T_PAR_left	0,59	0,04	0,62	0,03	2,30	0,73	0,06
T_PAR_right	0,58	0,04	0,60	0,03	1,43	0,45	0,19
T_PREM_left	0,61	0,04	0,63	0,02	1,58	0,50	0,17
T_PREM_right	0,62	0,04	0,63	0,02	1,37	0,43	0,21
UF_left	0,49	0,05	0,51	0,03	1,56	0,49	0,17
UF_right	0,48	0,04	0,49	0,02	1,45	0,46	0,19

Comparison of NDI in white matter fibers traversing through white matter lesions in NMOSD and whole tracts in HC

Tract	NMOSD		HC		T-value	Cohen's d	p-value (FDR corrected)
	M	***SD***	***M***	***SD***			
AF_left	0,61	0,06	0,64	0,04	1,48	0,61	0,24
AF_right	0,62	0,06	0,65	0,04	1,35	0,58	0,28
ATR_left	0,56	0,04	0,59	0,02	2,59	1,02	0,05
ATR_right	0,56	0,04	0,59	0,03	1,97	0,77	0,12
CC_1	0,53	0,05	0,56	0,04	2,03	0,88	0,12
CC_2	0,57	0,05	0,60	0,04	1,76	0,65	0,16
CC_3	0,62	0,04	0,64	0,04	0,91	0,34	0,45
CC_4	0,64	0,04	0,67	0,03	2,09	0,89	0,11
CC_5	0,61	0,04	0,65	0,03	2,61	1,20	0,06
CC_6	0,63	0,05	0,65	0,04	1,06	0,37	0,39
CC_7	0,64	0,03	0,65	0,03	1,40	0,48	0,26
CG_left	0,57	0,06	0,58	0,03	0,24	0,11	0,85
CG_right	0,56	0,06	0,59	0,03	1,60	0,83	0,22
CST_left	0,69	0,01	0,71	0,02	2,73	0,93	**0,04***
CST_right	0,68	0,03	0,72	0,02	3,12	1,36	**0,03***
FPT_left	0,66	0,04	0,67	0,02	1,04	0,40	0,39
FPT_right	0,65	0,04	0,68	0,02	1,87	0,80	0,15
ICP_left	0,68	0,01	0,72	0,03	2,88	1,09	0,15
IFO_left	0,53	0,05	0,56	0,03	2,71	0,92	0,09
IFO_right	0,51	0,05	0,55	0,03	3,51	1,33	**0,04***
ILF_left	0,52	0,04	0,56	0,03	4,16	1,55	**0,01****
ILF_right	0,49	0,04	0,55	0,03	0,44	0,17	**0,00****
MCP	0,75	0,01	0,75	0,02	2,18	0,73	0,74

OR_left	0,55	0,03	0,57	0,04	2,48	0,87	0,09
OR_right	0,54	0,04	0,57	0,03	2,22	0,96	0,05
POPT_left	0,64	0,04	0,67	0,03	2,16	0,85	0,10
POPT_right	0,64	0,03	0,66	0,02	-2,05	-1,22	0,10
SCP_left	0,71	0,01	0,69	0,02	2,30	1,16	0,32
SLF_III_left	0,60	0,05	0,65	0,04	2,43	1,04	0,11
SLF_III_right	0,62	0,06	0,65	0,04	2,48	1,15	0,39
SLF_II_left	0,63	0,04	0,67	0,03	1,68	0,68	0,07
SLF_II_right	0,62	0,05	0,67	0,03	2,70	1,23	0,07
SLF_I_left	0,62	0,06	0,65	0,03	4,89	2,13	0,18
SLF_I_right	0,61	0,04	0,66	0,03	3,60	1,65	0,06
STR_left	0,60	0,02	0,65	0,02	1,05	0,43	**0,00** **
STR_right	0,60	0,03	0,65	0,02	2,00	0,92	**0,02** *
ST_FO_left	0,53	0,05	0,54	0,03	2,02	0,82	0,39
ST_FO_right	0,50	0,05	0,54	0,03	1,87	0,78	0,13
ST_PREM_left	0,59	0,05	0,62	0,03	3,14	0,97	0,12
ST_PREM_right	0,60	0,05	0,63	0,03	2,15	0,77	0,15
T_OCC_left	0,54	0,02	0,57	0,04	4,94	1,69	**0,02** *
T_OCC_right	0,54	0,04	0,57	0,03	1,62	0,59	0,09
T_PAR_left	0,57	0,03	0,62	0,03	1,73	0,83	**0,00** ***
T_PAR_right	0,58	0,04	0,60	0,03	1,46	0,70	0,19
T_PREM_left	0,60	0,04	0,63	0,02	0,23	0,12	0,18
T_PREM_right	0,61	0,05	0,63	0,02	1,36	0,80	0,26
UF_left	0,51	0,06	0,51	0,03	1,54	0,49	0,86
UF_right	0,47	0,06	0,49	0,02	1,51	0,48	0,30

Comparison of NDI in white matter fibers not traversing through white matter lesions in NMOSD and whole tracts in HC

Tract	NMOSD		HC		T-value	Cohen's d	p-value (FDR corrected)
	M	*SD*	*M*	*SD*			
AF_left	0,62	0,06	0,64	0,04	1,29	0,41	0,28
AF_right	0,63	0,05	0,65	0,04	1,40	0,44	0,24
ATR_left	0,58	0,04	0,59	0,02	1,09	0,35	0,35
ATR_right	0,58	0,04	0,59	0,03	0,84	0,27	0,45
CC_1	0,55	0,05	0,56	0,04	1,07	0,34	0,36
CC_2	0,58	0,05	0,60	0,04	1,40	0,44	0,24
CC_3	0,62	0,05	0,64	0,04	1,25	0,40	0,29
CC_4	0,65	0,04	0,67	0,03	1,74	0,58	0,17
CC_5	0,63	0,04	0,65	0,03	1,61	0,51	0,19
CC_6	0,62	0,05	0,65	0,04	1,63	0,52	0,19
CC_7	0,63	0,04	0,65	0,03	1,64	0,52	0,19
CG_left	0,56	0,05	0,58	0,03	1,43	0,45	0,24

CG_right	0,57	0,05	0,59	0,03	1,47	0,47	0,23
CST_left	0,70	0,03	0,71	0,02	1,00	0,32	0,38
CST_right	0,70	0,03	0,72	0,02	1,55	0,49	0,21
FPT_left	0,66	0,04	0,67	0,02	1,40	0,44	0,24
FPT_right	0,66	0,03	0,68	0,02	1,23	0,39	0,29
ICP_left	0,71	0,03	0,72	0,03	0,22	0,07	0,84
ICP_right	0,70	0,04	0,71	0,03	0,32	0,10	0,78
IFO_left	0,54	0,04	0,56	0,03	1,23	0,39	0,29
IFO_right	0,54	0,04	0,55	0,03	1,26	0,40	0,29
ILF_left	0,54	0,05	0,56	0,03	1,78	0,56	0,16
ILF_right	0,53	0,05	0,55	0,03	1,94	0,61	0,13
MCP	0,75	0,02	0,75	0,02	0,26	0,08	0,81
OR_left	0,56	0,04	0,57	0,04	0,90	0,28	0,43
OR_right	0,56	0,03	0,57	0,03	0,67	0,22	0,55
POPT_left	0,67	0,03	0,67	0,03	0,58	0,18	0,59
POPT_right	0,66	0,02	0,66	0,02	0,86	0,27	0,45
SCP_left	0,68	0,03	0,69	0,02	0,85	0,27	0,45
SCP_right	0,69	0,03	0,69	0,02	0,02	0,01	0,98
SLF_III_left	0,63	0,05	0,65	0,04	1,41	0,45	0,24
SLF_III_right	0,63	0,05	0,65	0,04	1,22	0,39	0,29
SLF_II_left	0,64	0,05	0,67	0,03	1,96	0,62	0,13
SLF_II_right	0,65	0,04	0,67	0,03	1,61	0,51	0,19
SLF_I_left	0,63	0,04	0,65	0,03	1,56	0,49	0,21
SLF_I_right	0,64	0,04	0,66	0,03	1,85	0,58	0,15
STR_left	0,64	0,04	0,65	0,02	1,41	0,46	0,24
STR_right	0,63	0,03	0,65	0,02	1,42	0,45	0,24
ST_FO_left	0,52	0,05	0,54	0,03	1,67	0,53	0,19
ST_FO_right	0,52	0,05	0,54	0,03	1,34	0,42	0,26
ST_PREM_left	0,61	0,04	0,62	0,03	1,00	0,32	0,38
ST_PREM_right	0,62	0,04	0,63	0,03	1,22	0,39	0,29
T_OCC_left	0,56	0,04	0,57	0,04	0,69	0,22	0,55
T_OCC_right	0,56	0,03	0,57	0,03	0,63	0,20	0,57
T_PAR_left	0,61	0,04	0,62	0,03	1,07	0,34	0,36
T_PAR_right	0,59	0,03	0,60	0,03	0,72	0,23	0,53
T_PREM_left	0,61	0,04	0,63	0,02	1,46	0,46	0,24
T_PREM_right	0,62	0,04	0,63	0,02	1,13	0,36	0,33
UF_left	0,49	0,05	0,51	0,03	1,54	0,49	0,22
UF_right	0,48	0,04	0,49	0,02	1,51	0,48	0,22

p < ,05, **p < 0,01, ***p < ,001; NMOSD = neuromyelitis optica spectrum disorders; HC = healthy controls; NDI = neurite density index; AF = arcuate fascicle; ATR = anterior thalamic radiation; CC = corpus callosum; CC_1 = rostrum; CC_2 = genu; CC_3 = rostral body; CC_4 = anterior midbody; CC_5 = posterior midbody; CC_6 = isthmus; CC_7 = splenium; CG = cingulum; CST = corticospinal tract; FPT = fronto-pontine tract; ICP = inferior cerebellar peduncle; IFO = inferior occipito-frontal fascicle; ILF = inferior longitudinal fascicle; MCP = middle cerebellar peduncle; OR = optic radiation; POPT = parieto-occipital pontine tract; SCP = superior cerebellar peduncle; SLF_I = superior longitudinal fascicle I; SLF_II = superior longitudinal fascicle II; SLF_III = superior longitudinal fascicle III; STR = superior thalamic radiation; ST_FO = striato-fronto-orbital; ST_PREM = striato-premotor; T_OCC = thalamo-occipital; T_PAR = thalamo-parietal; T_PREM = thalamo-premotor; UF = uncinate fascicle.

Supplementary Table 4

Comparison of T1 relaxation rates in cerebral cortex between MS and HC

Parcellation	MS		HC		T-value	Cohen's d	p-value (FDR corrected)
	M	***SD***	***M***	***SD***			
lh_unknown	0.17936	0.01850	0.18141	0.01607	-0,35	-0,12	0,80
lh_Fronto-marginal gyrus (of Wernicke) and sulcus	0.67448	0.01154	0.69032	0.01815	-3,21	-1,02	**0,02***
lh_Inferior occipital gyrus (o3) and sulcus	0.73172	0.02061	0.74479	0.02002	-1,95	-0,64	0,14
lh_paracentral lobule and sulcus	0.73887	0.01895	0.75661	0.02392	-2,52	-0,81	0,05
lh_subcentral gyrus (central operculum) and sulci	0.69975	0.01263	0.72055	0.00932	-5,62	-1,90	**0,00****
lh_transverse frontopolar gyri and sulci	0.65609	0.01341	0.67214	0.01799	-3,10	-1,00	**0,02***
lh_anterior part of the cingulate gyrus and sulcus (aCC)	0.67624	0.00752	0.69083	0.01752	-3,38	-1,05	**0,01***
lh_middle-anterior part of the cingulate gyrus and sulcus (amCC)	0.68864	0.01368	0.70666	0.01942	-3,30	-1,06	**0,01***
lh_middle-posterior part of the cingulate gyrus and sulcus (pmCC)	0.70532	0.01936	0.72393	0.01730	-3,06	-1,02	**0,02***
lh_posterior-dorsal part of the cingulate gyrus (dpCC)	0.72234	0.01218	0.73916	0.01794	-3,38	-1,08	**0,01***
lh_posterior-ventral part of the cingulate gyrus (vpCC, isthmus of the cingulate gyrus)	0.74569	0.01374	0.76185	0.02021	-2,88	-0,92	**0,03***
lh_Cuneus (o6)	0.73493	0.01627	0.74950	0.01287	-2,98	-1,00	**0,02***
lh_opercular part of the inferior frontal gyrus	0.68544	0.01031	0.70444	0.01263	-5,04	-1,63	**0,00****
lh_orbital part of the inferior frontal gyrus	0.67914	0.01516	0.69592	0.01479	-3,39	-1,12	**0,01***
lh_triangular part of the inferior frontal gyrus	0.68052	0.01429	0.69400	0.01411	-2,88	-0,95	**0,03***
lh_middle frontal gyrus (F2)	0.66731	0.01050	0.67830	0.01346	-2,79	-0,90	**0,03***
lh_superior frontal gyrus (F1)	0.68053	0.01207	0.69350	0.01443	-2,98	-0,97	**0,02***
lh_long insular gyrus and central sulcus of the insula	0.67885	0.01392	0.69806	0.01469	-4,08	-1,34	**0,00****
lh_short insular gyri	0.65912	0.00910	0.67534	0.01420	-4,20	-1,34	**0,00****
lh_middle occipital gyrus (o2, lateral occipital gyrus)	0.71172	0.01478	0.72510	0.01303	-2,90	-0,97	**0,03***

lh_superior occipital gyrus (o1)	0.72018	0.01756	0.73777	0.01053	-3,61	-1,24	**0,01***
lh_lateral occipito-temporal gyrus (fusiform gyrus, o4-t4)	0.71912	0.01527	0.73276	0.01068	-3,09	-1,05	**0,02***
lh_lingual gyrus, lingual part of the medial occipito-temporal gyrus, (o5)	0.73499	0.01631	0.74591	0.01530	-2,09	-0,69	0,11
lh_parahippocampal gyrus, parahippocampal part of the medial occipito-temporal gyrus, (t5)	0.69684	0.01554	0.72301	0.02068	-4,39	-1,41	**0,00****
lh_orbital gyri	0.70025	0.01416	0.72031	0.01760	-3,84	-1,24	**0,01****
lh_angular gyrus	0.68392	0.01100	0.70055	0.01029	-4,72	-1,57	**0,00****
lh_supramarginal gyrus	0.68789	0.01155	0.70534	0.00744	-5,36	-1,83	**0,00****
lh_superior parietal lobule (lateral part of p1)	0.69367	0.01365	0.71176	0.01515	-3,82	-1,25	**0,01****
lh_postcentral gyrus	0.70855	0.01742	0.72892	0.01366	-3,91	-1,32	**0,01****
lh_precentral gyrus	0.72708	0.01654	0.74018	0.02192	-2,07	-0,67	0,11
lh_precuneus (medial part of p1)	0.70643	0.00892	0.72235	0.01269	-4,46	-1,43	**0,00****
lh_straight gyrus, gyrus rectus	0.68529	0.01046	0.69905	0.01918	-2,76	-0,87	**0,04***
lh_subcallosal area, subcallosal gyrus	0.64024	0.07068	0.68654	0.03269	-2,49	-0,87	0,06
lh_anterior transverse temporal gyrus (of heschl)	0.74462	0.02050	0.75563	0.02157	-1,59	-0,52	0,22
lh_lateral aspect of the superior temporal gyrus	0.69164	0.01396	0.70625	0.01185	-3,40	-1,14	**0,01***
lh_planum polare of the superior temporal gyrus	0.67115	0.01476	0.68271	0.02121	-1,95	-0,62	0,14
lh_planum temporale or temporal plane of the superior temporal gyrus	0.69971	0.01673	0.71206	0.01792	-2,17	-0,71	0,10
lh_Inferior temporal gyrus (t3)	0.68686	0.01547	0.70536	0.01264	-3,94	-1,32	**0,00****
lh_middle temporal gyrus (t2)	0.68208	0.01013	0.69737	0.00961	-4,68	-1,55	**0,00****
lh_horizontal ramus of the anterior segment of the lateral sulcus (or fissure)	0.70479	0.01340	0.73142	0.02303	-4,37	-1,38	**0,00****
lh_vertical ramus of the anterior segment of the lateral sulcus (or fissure)	0.70529	0.01737	0.73023	0.02020	-4,04	-1,32	**0,00****
lh_posterior ramus (or segment) of the lateral sulcus (or fissure)	0.72861	0.01546	0.73981	0.01869	-1,99	-0,65	0,12
lh_occipital pole	0.73809	0.01756	0.75472	0.02035	-2,67	-0,87	**0,04***
lh_temporal pole	0.67477	0.01085	0.69383	0.02546	-3,04	-0,95	**0,02***

lh_Calcarine sulcus	0.73564	0.01591	0.75114	0.01649	-2,91	-0,96	**0,03***
lh_Central sulcus (Rolando's fissure)	0.74204	0.01447	0.76043	0.01448	-3,85	-1,27	**0,01****
lh_marginal sulcus of cingulate gyrus	0.70202	0.01903	0.72174	0.01264	-3,64	-1,24	**0,01****
lh_anterior circular sulcus of the insula	0.68486	0.01223	0.70433	0.01699	-4,04	-1,30	**0,00****
lh_Inferior circular sulcus of the insula	0.68801	0.01227	0.70246	0.01700	-2,99	-0,96	**0,02***
lh_superior circular sulcus of the insula	0.70022	0.01002	0.71905	0.01349	-4,86	-1,56	**0,00****
lh_anterior transverse collateral sulcus	0.68337	0.01702	0.71009	0.01906	-4,50	-1,47	**0,00****
lh_posterior transverse collateral sulcus	0.74995	0.02451	0.76334	0.03286	-1,42	-0,46	0,27
lh_Inferior frontal sulcus	0.70204	0.01364	0.71520	0.01258	-3,03	-1,01	**0,02***
lh_middle frontal sulcus	0.67529	0.01167	0.69399	0.01368	-4,48	-1,46	**0,00****
lh_superior frontal sulcus	0.68534	0.01049	0.70444	0.01289	-4,97	-1,61	**0,00****
lh_Intermediate sulcus of Jensen	0.68899	0.01463	0.69903	0.02022	-1,75	-0,56	0,18
lh_Intraparietal sulcus and posterior transverse parietal sulcus	0.71367	0.01245	0.73262	0.01340	-4,46	-1,46	**0,00****
lh_middle occipital sulcus and sulcus lunatus (secondary intermediate sulcus)	0.71891	0.01489	0.73550	0.02893	-2,24	-0,70	0,09
lh_superior occipital sulcus and transverse occipital sulcus (superior intermediate sulcus)	0.72340	0.01500	0.73855	0.01810	-2,78	-0,90	**0,03***
lh_anterior occipital sulcus	0.72081	0.01929	0.74230	0.03259	-2,48	-0,79	0,06
lh_lateral occipito-temporal sulcus	0.70828	0.01815	0.72408	0.02152	-2,42	-0,79	0,06
lh_medial occipito-temporal sulcus and lingual sulcus	0.71500	0.01633	0.73984	0.01645	-4,60	-1,52	**0,00****
lh_lateral orbital sulcus	0.67609	0.01498	0.68656	0.01628	-2,03	-0,67	0,12
lh_medial orbital sulcus, olfactory sulcus	0.70981	0.01086	0.73586	0.02364	-4,41	-1,38	**0,00****
lh_h-shaped orbital sulcus	0.70873	0.01542	0.73057	0.01846	-3,92	-1,27	**0,00****
lh_parieto-occipital sulcus	0.73912	0.00944	0.75240	0.01520	-3,24	-1,03	**0,02***
lh_pericallosal sulcus	0.73907	0.04294	0.77760	0.02894	-3,14	-1,07	**0,02***
lh_postcentral sulcus	0.71074	0.01167	0.72634	0.01504	-3,55	-1,15	**0,01****
lh_Inferior part of the precentral sulcus	0.70024	0.00830	0.71509	0.01351	-4,09	-1,30	**0,01****

lh_superior part of the precentral sulcus	0.71419	0.01214	0.72951	0.01764	-3,11	-1,00	**0,02***
lh_suborbital sulcus	0.68078	0.00987	0.69764	0.02038	-3,27	-1,03	**0,02***
lh_subparietal sulcus	0.71819	0.01133	0.73345	0.01413	-3,65	-1,18	**0,01****
lh_Inferior temporal sulcus	0.69451	0.01279	0.71024	0.01215	-3,81	-1,26	**0,01****
lh_superior temporal sulcus	0.70438	0.01160	0.71564	0.00838	-3,33	-1,13	**0,01***
lh_transverse temporal sulcus (heschl's gyrus)	0.73535	0.02174	0.74877	0.02307	-1,82	-0,60	0,16
rh_unknown	0.16848	0.01452	0.17213	0.01981	-0,65	-0,21	0,62
rh_Fronto-marginal gyrus (of Wernicke) and sulcus	0.67384	0.01283	0.68476	0.02013	-2,00	-0,64	0,12
rh_Inferior occipital gyrus (o3) and sulcus	0.74189	0.02307	0.75208	0.02062	-1,41	-0,47	0,28
rh_paracentral lobule and sulcus	0.73958	0.01315	0.76494	0.01835	-4,88	-1,57	**0,00****
rh_subcentral gyrus (central operculum) and sulci	0.71811	0.01664	0.73418	0.01751	-2,86	-0,94	**0,03***
rh_transverse frontopolar gyri and sulci	0.65464	0.01149	0.66620	0.01871	-2,30	-0,73	0,08
rh_anterior part of the cingulate gyrus and sulcus (aCC)	0.67273	0.00965	0.69086	0.01364	-4,71	-1,51	**0,00****
rh_middle-anterior part of the cingulate gyrus and sulcus (amCC)	0.69436	0.01058	0.71125	0.01572	-3,88	-1,24	**0,01****
rh_middle-posterior part of the cingulate gyrus and sulcus (pmCC)	0.72020	0.00864	0.73653	0.01393	-4,35	-1,38	**0,00****
rh_posterior-dorsal part of the cingulate gyrus (dpCC)	0.73079	0.01021	0.74712	0.01484	-3,94	-1,26	**0,00****
rh_posterior-ventral part of the cingulate gyrus (vpCC, isthmus of the cingulate gyrus)	0.75964	0.03107	0.77931	0.02033	-2,23	-0,76	0,09
rh_Cuneus (o6)	0.74314	0.01774	0.75519	0.01410	-2,26	-0,76	0,08
rh_opercular part of the inferior frontal gyrus	0.69359	0.01080	0.71079	0.01773	-3,62	-1,15	**0,01****
rh_orbital part of the inferior frontal gyrus	0.68158	0.01517	0.69537	0.01838	-2,50	-0,81	0,06
rh_triangular part of the inferior frontal gyrus	0.68662	0.01307	0.69754	0.01685	-2,22	-0,72	0,09
rh_middle frontal gyrus (F2)	0.67098	0.01011	0.68300	0.01238	-3,25	-1,05	**0,01***
rh_superior frontal gyrus (F1)	0.67681	0.00944	0.69483	0.01374	-4,70	-1,51	**0,00****
rh_long insular gyrus and central sulcus of the insula	0.67021	0.01161	0.68168	0.01495	-2,62	-0,85	**0,05***
rh_short insular gyri	0.67135	0.01202	0.68019	0.01684	-1,85	-0,60	0,16

rh_middle occipital gyrus (o2, lateral occipital gyrus)	0.71315	0.00980	0.72610	0.01081	-3,82	-1,25	**0,01****
rh_superior occipital gyrus (o1)	0.72724	0.01351	0.74714	0.01153	-4,77	-1,60	**0,00****
rh_lateral occipito-temporal gyrus (fusiform gyrus, o4-t4)	0.75536	0.01412	0.76545	0.01932	-1,83	-0,59	0,16
rh_lingual gyrus, lingual part of the medial occipito-temporal gyrus, (o5)	0.75218	0.01610	0.76080	0.01756	-1,56	-0,51	0,23
rh_parahippocampal gyrus, parahippocampal part of the medial occipito-temporal gyrus, (t5)	0.71802	0.01294	0.73839	0.02291	-3,39	-1,07	**0,01***
rh_orbital gyri	0.68616	0.01083	0.70545	0.01953	-3,79	-1,20	**0,01****
rh_angular gyrus	0.69831	0.01049	0.70989	0.01088	-3,29	-1,08	**0,01***
rh_supramarginal gyrus	0.70146	0.01201	0.71135	0.01223	-2,47	-0,82	0,06
rh_superior parietal lobule (lateral part of p1)	0.70247	0.01059	0.72037	0.01463	-4,30	-1,38	0,00
rh_postcentral gyrus	0.72389	0.01964	0.73948	0.01580	-2,63	-0,88	0,05
rh_precentral gyrus	0.73838	0.01343	0.75416	0.02001	-2,85	-0,91	**0,03***
rh_precuneus (medial part of p1)	0.71568	0.01156	0.72993	0.01444	-3,33	-1,08	**0,01***
rh_straight gyrus, gyrus rectus	0.69134	0.01166	0.70339	0.01857	-2,40	-0,76	0,07
rh_subcallosal area, subcallosal gyrus	0.58262	0.10558	0.63242	0.10239	-1,45	-0,48	0,27
rh_anterior transverse temporal gyrus (of heschl)	0.75789	0.02135	0.76557	0.02426	-1,02	-0,33	0,43
rh_lateral aspect of the superior temporal gyrus	0.70751	0.01491	0.71940	0.01627	-2,32	-0,76	0,08
rh_planum polare of the superior temporal gyrus	0.67672	0.01520	0.69346	0.02209	-2,72	-0,87	**0,04***
rh_planum temporale or temporal plane of the superior temporal gyrus	0.71839	0.02103	0.73162	0.01818	-2,03	-0,68	0,12
rh_Inferior temporal gyrus (t3)	0.71624	0.01318	0.72906	0.01619	-2,65	-0,86	**0,04***
rh_middle temporal gyrus (t2)	0.68529	0.01173	0.69705	0.00990	-3,26	-1,09	**0,01***
rh_horizontal ramus of the anterior segment of the lateral sulcus (or fissure)	0.71695	0.01453	0.74428	0.02598	-4,02	-1,27	**0,00****
rh_vertical ramus of the anterior segment of the lateral sulcus (or fissure)	0.71442	0.01927	0.72382	0.01996	-1,46	-0,48	0,26
rh_posterior ramus (or segment) of the lateral sulcus (or fissure)	0.73656	0.01146	0.74428	0.01690	-1,65	-0,53	0,20
rh_occipital pole	0.74634	0.01695	0.76007	0.01761	-2,41	-0,79	0,06

rh_temporal pole	0.68972	0.01449	0.70443	0.01987	-2,60	-0,84	**0,05***
rh_Calcarine sulcus	0.75094	0.01597	0.76601	0.01836	-2,67	-0,87	**0,04***
rh_Central sulcus (Rolando's fissure)	0.74783	0.01465	0.76958	0.01321	-4,70	-1,57	**0,00****
rh_marginal sulcus of cingulate gyrus	0.71986	0.01179	0.73632	0.01336	-3,98	-1,30	**0,00****
rh_anterior circular sulcus of the insula	0.68516	0.01188	0.70057	0.02092	-2,80	-0,89	**0,03***
rh_Inferior circular sulcus of the insula	0.69528	0.01314	0.70295	0.01869	-1,46	-0,47	0,26
rh_superior circular sulcus of the insula	0.70005	0.01248	0.71489	0.01759	-2,99	-0,96	**0,02***
rh_anterior transverse collateral sulcus	0.73145	0.01724	0.74730	0.01947	-2,63	-0,86	**0,05***
rh_posterior transverse collateral sulcus	0.77674	0.02738	0.77911	0.03641	-0,23	-0,07	0,86
rh_Inferior frontal sulcus	0.71042	0.01584	0.72281	0.01709	-2,28	-0,75	0,08
rh_middle frontal sulcus	0.67742	0.01065	0.69099	0.01367	-3,39	-1,10	**0,01***
rh_superior frontal sulcus	0.68884	0.01059	0.70534	0.01617	-3,72	-1,19	**0,01****
rh_Intermediate sulcus of Jensen	0.70200	0.01995	0.72261	0.02515	-2,78	-0,90	**0,03***
rh_Intraparietal sulcus and posterior transverse parietal sulcus	0.72643	0.01074	0.74182	0.01141	-4,22	-1,38	**0,00****
rh_middle occipital sulcus and sulcus lunatus (secondary intermediate sulcus)	0.72983	0.01803	0.74145	0.02037	-1,84	-0,60	0,16
rh_superior occipital sulcus and transverse occipital sulcus (superior intermediate sulcus)	0.73618	0.01222	0.74772	0.01040	-3,06	-1,02	**0,02***
rh_anterior occipital sulcus	0.73519	0.01608	0.74572	0.02219	-1,67	-0,54	0,20
rh_lateral occipito-temporal sulcus	0.75357	0.01880	0.76231	0.02168	-1,31	-0,43	0,31
rh_medial occipito-temporal sulcus and lingual sulcus	0.74359	0.01648	0.76333	0.02005	-3,29	-1,07	**0,01***
rh_lateral orbital sulcus	0.67978	0.01529	0.69364	0.01621	-2,67	-0,88	**0,04***
rh_medial orbital sulcus, olfactory sulcus	0.68782	0.01620	0.71715	0.03229	-3,57	-1,12	**0,01****
rh_h-shaped orbital sulcus	0.69404	0.01155	0.71893	0.02208	-4,38	-1,38	**0,00****
rh_parieto-occipital sulcus	0.75702	0.01224	0.76481	0.01527	-1,72	-0,56	0,18
rh_pericallosal sulcus	0.71968	0.06646	0.77785	0.03032	-3,33	-1,16	**0,02***
rh_postcentral sulcus	0.72898	0.01801	0.74263	0.01759	-2,32	-0,77	0,08

rh_Inferior part of the precentral sulcus	0.70414	0.01224	0.71869	0.01425	-3,34	-1,09	**0,01***
rh_superior part of the precentral sulcus	0.71716	0.01145	0.73651	0.01964	-3,72	-1,18	**0,01****
rh_suborbital sulcus	0.67372	0.01091	0.68825	0.01952	-2,85	-0,90	**0,03***
rh_subparietal sulcus	0.72403	0.00912	0.73966	0.01473	-3,94	-1,25	**0,00****
rh_Inferior temporal sulcus	0.70945	0.01508	0.72076	0.01646	-2,18	-0,71	0,09
rh_superior temporal sulcus	0.71848	0.01096	0.73014	0.01114	-3,20	-1,05	**0,02***
rh_transverse temporal sulcus (heschl's gyrus)	0.74698	0.01477	0.75979	0.02426	-1,97	-0,63	0,13

Comparison of T1 relaxation rates in cerebral cortex between NMOSD and HC

Parcellation	NMOSD		HC		T-value	Cohen's d	p-value (FDR corrected)
	M	*SD*	*M*	*SD*			
lh_unknown	0.17427	0.01541	0.18141	0.01607	-1,43	-0,45	0,27
lh_Fronto-marginal gyrus (of Wernicke) and sulcus	0.68286	0.03243	0.69032	0.01815	-0,90	-0,28	0,49
lh_Inferior occipital gyrus (o3) and sulcus	0.75060	0.03183	0.74479	0.02002	0,69	0,22	0,60
lh_paracentral lobule and sulcus	0.74781	0.02140	0.75661	0.02392	-1,23	-0,39	0,34
lh_subcentral gyrus (central operculum) and sulci	0.72223	0.01902	0.72055	0.00932	0,35	0,11	0,80
lh_transverse frontopolar gyri and sulci	0.66443	0.02949	0.67214	0.01799	-1,00	-0,32	0,44
lh_anterior part of the cingulate gyrus and sulcus (aCC)	0.68404	0.01794	0.69083	0.01752	-1,21	-0,38	0,35
lh_middle-anterior part of the cingulate gyrus and sulcus (amCC)	0.69638	0.02080	0.70666	0.01942	-1,62	-0,51	0,21
lh_middle-posterior part of the cingulate gyrus and sulcus (pmCC)	0.71478	0.01875	0.72393	0.01730	-1,60	-0,51	0,21
lh_posterior-dorsal part of the cingulate gyrus (dpCC)	0.73379	0.02327	0.73916	0.01794	-0,82	-0,26	0,53
lh_posterior-ventral part of the cingulate gyrus (vpCC, isthmus of the cingulate gyrus)	0.76949	0.03157	0.76185	0.02021	0,91	0,29	0,49
lh_Cuneus (o6)	0.73950	0.02748	0.74950	0.01287	-1,47	-0,47	0,26
lh_opercular part of the inferior frontal gyrus	0.69852	0.01605	0.70444	0.01263	-1,30	-0,41	0,31
lh_orbital part of the inferior frontal gyrus	0.69354	0.02026	0.69592	0.01479	-0,43	-0,13	0,76
lh_triangular part of the inferior frontal gyrus	0.69276	0.01924	0.69400	0.01411	-0,23	-0,07	0,85

lh_middle frontal gyrus (F2)	0.67678	0.01914	0.67830	0.01346	-0,29	-0,09	0,83
lh_superior frontal gyrus (F1)	0.68817	0.02015	0.69350	0.01443	-0,96	-0,30	0,46
lh_long insular gyrus and central sulcus of the insula	0.68893	0.01933	0.69806	0.01469	-1,68	-0,53	0,20
lh_short insular gyri	0.67120	0.01289	0.67534	0.01420	-0,97	-0,31	0,46
lh_middle occipital gyrus (o2, lateral occipital gyrus)	0.71981	0.02051	0.72510	0.01303	-0,97	-0,31	0,46
lh_superior occipital gyrus (o1)	0.72842	0.02439	0.73777	0.01053	-1,57	-0,50	0,23
lh_lateral occipito-temporal gyrus (fusiform gyrus, o4-t4)	0.74182	0.02630	0.73276	0.01068	1,43	0,45	0,27
lh_lingual gyrus, lingual part of the medial occipito-temporal gyrus, (o5)	0.75101	0.02215	0.74591	0.01530	0,85	0,27	0,51
lh_parahippocampal gyrus, parahippocampal part of the medial occipito-temporal gyrus, (t5)	0.71631	0.01884	0.72301	0.02068	-1,07	-0,34	0,41
lh_orbital gyri	0.72404	0.06246	0.72031	0.01760	0,26	0,08	0,85
lh_angular gyrus	0.69137	0.02183	0.70055	0.01029	-1,70	-0,54	0,19
lh_supramarginal gyrus	0.69991	0.01954	0.70534	0.00744	-1,16	-0,37	0,38
lh_superior parietal lobule (lateral part of p1)	0.69986	0.02305	0.71176	0.01515	-1,93	-0,61	0,14
lh_postcentral gyrus	0.71837	0.03727	0.72892	0.01366	-1,19	-0,38	0,37
lh_precentral gyrus	0.73803	0.02327	0.74018	0.02192	-0,30	-0,09	0,83
lh_precuneus (medial part of p1)	0.71291	0.02207	0.72235	0.01269	-1,66	-0,52	0,20
lh_straight gyrus, gyrus rectus	0.71660	0.08612	0.69905	0.01918	0,89	0,28	0,49
lh_subcallosal area, subcallosal gyrus	0.65983	0.05771	0.68654	0.03269	-1,80	-0,57	0,17
lh_anterior transverse temporal gyrus (of heschl)	0.74606	0.03618	0.75563	0.02157	-1,02	-0,32	0,44
lh_lateral aspect of the superior temporal gyrus	0.70398	0.01855	0.70625	0.01185	-0,46	-0,15	0,74
lh_planum polare of the superior temporal gyrus	0.67365	0.01919	0.68271	0.02121	-1,42	-0,45	0,27
lh_planum temporale or temporal plane of the superior temporal gyrus	0.69546	0.03571	0.71206	0.01792	-1,86	-0,59	0,16
lh_Inferior temporal gyrus (t3)	0.70696	0.02019	0.70536	0.01264	0,30	0,09	0,83
lh_middle temporal gyrus (t2)	0.69139	0.01842	0.69737	0.00961	-1,29	-0,41	0,32

lh_horizontal ramus of the anterior segment of the lateral sulcus (or fissure)	0.73672	0.02472	0.73142	0.02303	0,70	0,22	0,59
lh_vertical ramus of the anterior segment of the lateral sulcus (or fissure)	0.72081	0.01949	0.73023	0.02020	-1,50	-0,47	0,25
lh_posterior ramus (or segment) of the lateral sulcus (or fissure)	0.73730	0.02258	0.73981	0.01869	-0,38	-0,12	0,78
lh_occipital pole	0.75617	0.02731	0.75472	0.02035	0,19	0,06	0,88
lh_temporal pole	0.69091	0.02469	0.69383	0.02546	-0,37	-0,12	0,79
lh_Calcarine sulcus	0.75364	0.02790	0.75114	0.01649	0,34	0,11	0,80
lh_Central sulcus (Rolando's fissure)	0.74199	0.02512	0.76043	0.01448	-2,84	-0,90	**0,03***
lh_marginal sulcus of cingulate gyrus	0.71128	0.02263	0.72174	0.01264	-1,81	-0,57	0,17
lh_anterior circular sulcus of the insula	0.70352	0.02043	0.70433	0.01699	-0,14	-0,04	0,91
lh_Inferior circular sulcus of the insula	0.68839	0.02124	0.70246	0.01700	-2,31	-0,73	0,08
lh_superior circular sulcus of the insula	0.71803	0.01656	0.71905	0.01349	-0,21	-0,07	0,87
lh_anterior transverse collateral sulcus	0.71181	0.03010	0.71009	0.01906	0,22	0,07	0,86
lh_posterior transverse collateral sulcus	0.78360	0.04344	0.76334	0.03286	1,66	0,53	0,20
lh_Inferior frontal sulcus	0.71391	0.02056	0.71520	0.01258	-0,24	-0,08	0,85
lh_middle frontal sulcus	0.68273	0.01939	0.69399	0.01368	-2,12	-0,67	0,10
lh_superior frontal sulcus	0.69944	0.02098	0.70444	0.01289	-0,91	-0,29	0,49
lh_Intermediate sulcus of Jensen	0.70134	0.03523	0.69903	0.02022	0,25	0,08	0,85
lh_Intraparietal sulcus and posterior transverse parietal sulcus	0.72436	0.02153	0.73262	0.01340	-1,46	-0,46	0,26
lh_middle occipital sulcus and sulcus lunatus (secondary intermediate sulcus)	0.72653	0.02658	0.73550	0.02893	-1,02	-0,32	0,43
lh_superior occipital sulcus and transverse occipital sulcus (superior intermediate sulcus)	0.72306	0.02322	0.73855	0.01810	-2,35	-0,74	0,07
lh_anterior occipital sulcus	0.73293	0.03427	0.74230	0.03259	-0,89	-0,28	0,49
lh_lateral occipito-temporal sulcus	0.73391	0.04127	0.72408	0.02152	0,94	0,30	0,47
lh_medial occipito-temporal sulcus and lingual sulcus	0.74723	0.03118	0.73984	0.01645	0,94	0,30	0,47
lh_lateral orbital sulcus	0.69003	0.02164	0.68656	0.01628	0,57	0,18	0,67
lh_medial orbital sulcus, olfactory sulcus	0.72771	0.04445	0.73586	0.02364	-0,72	-0,23	0,58

lh_h-shaped orbital sulcus	0.72628	0.02525	0.73057	0.01846	-0,61	-0,19	0,64
lh_parieto-occipital sulcus	0.75029	0.02257	0.75240	0.01520	-0,35	-0,11	0,80
lh_pericallosal sulcus	0.77133	0.03272	0.77760	0.02894	-0,64	-0,20	0,63
lh_postcentral sulcus	0.72508	0.02532	0.72634	0.01504	-0,19	-0,06	0,88
lh_Inferior part of the precentral sulcus	0.70517	0.02113	0.71509	0.01351	-1,77	-0,56	0,17
lh_superior part of the precentral sulcus	0.72054	0.02379	0.72951	0.01764	-1,35	-0,43	0,29
lh_suborbital sulcus	0.69393	0.02847	0.69764	0.02038	-0,47	-0,15	0,73
lh_subparietal sulcus	0.72865	0.02287	0.73345	0.01413	-0,80	-0,25	0,54
lh_Inferior temporal sulcus	0.72020	0.02676	0.71024	0.01215	1,52	0,48	0,25
lh_superior temporal sulcus	0.71669	0.01708	0.71564	0.00838	0,25	0,08	0,85
lh_transverse temporal sulcus (heschl's gyrus)	0.73684	0.03171	0.74877	0.02307	-1,36	-0,43	0,29
rh_unknown	0.16215	0.01653	0.17213	0.01981	-1,73	-0,55	0,18
rh_Fronto-marginal gyrus (of Wernicke) and sulcus	0.69396	0.09622	0.68476	0.02013	0,42	0,13	0,77
rh_Inferior occipital gyrus (o3) and sulcus	0.75360	0.02943	0.75208	0.02062	0,19	0,06	0,88
rh_paracentral lobule and sulcus	0.75734	0.02388	0.76494	0.01835	-1,13	-0,36	0,39
rh_subcentral gyrus (central operculum) and sulci	0.72735	0.02032	0.73418	0.01751	-1,14	-0,36	0,38
rh_transverse frontopolar gyri and sulci	0.65782	0.03287	0.66620	0.01871	-0,99	-0,31	0,45
rh_anterior part of the cingulate gyrus and sulcus (aCC)	0.69117	0.01703	0.69086	0.01364	0,06	0,02	0,96
rh_middle-anterior part of the cingulate gyrus and sulcus (amCC)	0.71035	0.01507	0.71125	0.01572	-0,18	-0,06	0,88
rh_middle-posterior part of the cingulate gyrus and sulcus (pmCC)	0.73342	0.01625	0.73653	0.01393	-0,65	-0,21	0,62
rh_posterior-dorsal part of the cingulate gyrus (dpCC)	0.74337	0.01731	0.74712	0.01484	-0,74	-0,23	0,57
rh_posterior-ventral part of the cingulate gyrus (vpCC, isthmus of the cingulate gyrus)	0.78148	0.03021	0.77931	0.02033	0,27	0,08	0,84
rh_Cuneus (o6)	0.74530	0.02309	0.75519	0.01410	-1,63	-0,52	0,21
rh_opercular part of the inferior frontal gyrus	0.70254	0.01709	0.71079	0.01773	-1,50	-0,47	0,25
rh_orbital part of the inferior frontal gyrus	0.69237	0.01945	0.69537	0.01838	-0,50	-0,16	0,72

rh_triangular part of the inferior frontal gyrus	0.68880	0.01702	0.69754	0.01685	-1,63	-0,52	0,21
rh_middle frontal gyrus (F2)	0.67895	0.01605	0.68300	0.01238	-0,89	-0,28	0,49
rh_superior frontal gyrus (F1)	0.69216	0.01801	0.69483	0.01374	-0,53	-0,17	0,70
rh_long insular gyrus and central sulcus of the insula	0.67555	0.01220	0.68168	0.01495	-1,42	-0,45	0,27
rh_short insular gyri	0.67851	0.01449	0.68019	0.01684	-0,34	-0,11	0,81
rh_middle occipital gyrus (o2, lateral occipital gyrus)	0.72405	0.02078	0.72610	0.01081	-0,39	-0,12	0,78
rh_superior occipital gyrus (o1)	0.73296	0.02014	0.74714	0.01153	-2,73	-0,86	**0,04***
rh_lateral occipito-temporal gyrus (fusiform gyrus, o4-t4)	0.76629	0.02346	0.76545	0.01932	0,12	0,04	0,92
rh_lingual gyrus, lingual part of the medial occipito-temporal gyrus, (o5)	0.75843	0.02396	0.76080	0.01756	-0,36	-0,11	0,80
rh_parahippocampal gyrus, parahippocampal part of the medial occipito-temporal gyrus, (t5)	0.72893	0.01950	0.73839	0.02291	-1,41	-0,44	0,28
rh_orbital gyri	0.70892	0.09492	0.70545	0.01953	0,16	0,05	0,90
rh_angular gyrus	0.70178	0.01639	0.70989	0.01088	-1,84	-0,58	0,16
rh_supramarginal gyrus	0.70429	0.01679	0.71135	0.01223	-1,52	-0,48	0,24
rh_superior parietal lobule (lateral part of p1)	0.70309	0.02679	0.72037	0.01463	-2,53	-0,80	0,05
rh_postcentral gyrus	0.72738	0.02147	0.73948	0.01580	-2,03	-0,64	0,12
rh_precentral gyrus	0.75160	0.02218	0.75416	0.02001	-0,38	-0,12	0,78
rh_precuneus (medial part of p1)	0.72574	0.02087	0.72993	0.01444	-0,74	-0,23	0,57
rh_straight gyrus, gyrus rectus	0.72725	0.12620	0.70339	0.01857	0,84	0,26	0,52
rh_subcallosal area, subcallosal gyrus	0.60313	0.08010	0.63242	0.10239	-1,01	-0,32	0,44
rh_anterior transverse temporal gyrus (of heschl)	0.77162	0.02578	0.76557	0.02426	0,76	0,24	0,56
rh_lateral aspect of the superior temporal gyrus	0.71666	0.01855	0.71940	0.01627	-0,50	-0,16	0,72
rh_planum polare of the superior temporal gyrus	0.69178	0.01767	0.69346	0.02209	-0,27	-0,08	0,84
rh_planum temporale or temporal plane of the superior temporal gyrus	0.72626	0.01950	0.73162	0.01818	-0,90	-0,28	0,49
rh_Inferior temporal gyrus (t3)	0.72257	0.02191	0.72906	0.01619	-1,06	-0,34	0,42

rh_middle temporal gyrus (t2)	0.69596	0.01609	0.69705	0.00990	-0,26	-0,08	0,85
rh_horizontal ramus of the anterior segment of the lateral sulcus (or fissure)	0.73032	0.02194	0.74428	0.02598	-1,84	-0,58	0,16
rh_vertical ramus of the anterior segment of the lateral sulcus (or fissure)	0.71179	0.02542	0.72382	0.01996	-1,66	-0,53	0,20
rh_posterior ramus (or segment) of the lateral sulcus (or fissure)	0.74135	0.02157	0.74428	0.01690	-0,48	-0,15	0,73
rh_occipital pole	0.75539	0.02480	0.76007	0.01761	-0,69	-0,22	0,60
rh_temporal pole	0.70247	0.01880	0.70443	0.01987	-0,32	-0,10	0,81
rh_Calcarine sulcus	0.76116	0.02277	0.76601	0.01836	-0,74	-0,23	0,57
rh_Central sulcus (Rolando's fissure)	0.75165	0.02073	0.76958	0.01321	-3,26	-1,03	**0,01***
rh_marginal sulcus of cingulate gyrus	0.72585	0.01649	0.73632	0.01336	-2,21	-0,70	0,09
rh_anterior circular sulcus of the insula	0.69436	0.01624	0.70057	0.02092	-1,05	-0,33	0,42
rh_Inferior circular sulcus of the insula	0.70520	0.01790	0.70295	0.01869	0,39	0,12	0,78
rh_superior circular sulcus of the insula	0.70711	0.01777	0.71489	0.01759	-1,39	-0,44	0,28
rh_anterior transverse collateral sulcus	0.74155	0.02332	0.74730	0.01947	-0,85	-0,27	0,51
rh_posterior transverse collateral sulcus	0.78881	0.03453	0.77911	0.03641	0,86	0,27	0,50
rh_Inferior frontal sulcus	0.71438	0.02166	0.72281	0.01709	-1,37	-0,43	0,29
rh_middle frontal sulcus	0.67907	0.01919	0.69099	0.01367	-2,26	-0,72	0,08
rh_superior frontal sulcus	0.69988	0.02173	0.70534	0.01617	-0,90	-0,28	0,49
rh_Intermediate sulcus of Jensen	0.70622	0.02603	0.72261	0.02515	-2,02	-0,64	0,12
rh_Intraparietal sulcus and posterior transverse parietal sulcus	0.72888	0.02185	0.74182	0.01141	-2,35	-0,74	0,08
rh_middle occipital sulcus and sulcus lunatus (secondary intermediate sulcus)	0.73745	0.02498	0.74145	0.02037	-0,56	-0,18	0,68
rh_superior occipital sulcus and transverse occipital sulcus (superior intermediate sulcus)	0.73493	0.01940	0.74772	0.01040	-2,60	-0,82	**0,05***
rh_anterior occipital sulcus	0.74327	0.02560	0.74572	0.02219	-0,32	-0,10	0,81
rh_lateral occipito-temporal sulcus	0.76357	0.03393	0.76231	0.02168	0,14	0,04	0,91
rh_medial occipito-temporal sulcus and lingual sulcus	0.76287	0.02685	0.76333	0.02005	-0,06	-0,02	0,96
rh_lateral orbital sulcus	0.68415	0.01875	0.69364	0.01621	-1,71	-0,54	0,19

rh_medial orbital sulcus, olfactory sulcus	0.70622	0.06759	0.71715	0.03229	-0,65	-0,21	0,62
rh_h-shaped orbital sulcus	0.70674	0.04108	0.71893	0.02208	-1,17	-0,37	0,37
rh_parieto-occipital sulcus	0.76202	0.01950	0.76481	0.01527	-0,50	-0,16	0,72
rh_pericallosal sulcus	0.77529	0.02617	0.77785	0.03032	-0,29	-0,09	0,83
rh_postcentral sulcus	0.73765	0.02105	0.74263	0.01759	-0,81	-0,26	0,53
rh_Inferior part of the precentral sulcus	0.71447	0.01850	0.71869	0.01425	-0,81	-0,26	0,53
rh_superior part of the precentral sulcus	0.72095	0.02208	0.73651	0.01964	-2,36	-0,74	0,07
rh_suborbital sulcus	0.69935	0.05341	0.68825	0.01952	0,87	0,28	0,50
rh_subparietal sulcus	0.73569	0.01985	0.73966	0.01473	-0,72	-0,23	0,59
rh_Inferior temporal sulcus	0.72246	0.02317	0.72076	0.01646	0,27	0,08	0,84
rh_superior temporal sulcus	0.72620	0.01660	0.73014	0.01114	-0,88	-0,28	0,49
rh_transverse temporal sulcus (heschl's gyrus)	0.76004	0.02253	0.75979	0.02426	0,03	0,01	0,97

Comparison of T1 relaxation rates in cerebral cortex between MS and NMOSD

Parcellation	MS		NMOSD		T-value	Cohen's d	p-value (FDR corrected)
	M	*SD*	*M*	*SD*			
lh_unknown	0.17936	0.01850	0.17427	0.01541	-0,90	-0,30	0,49
lh_Fronto-marginal gyrus (of Wernicke) and sulcus	0.67448	0.01154	0.68286	0.03243	1,08	0,33	0,41
lh_Inferior occipital gyrus (o3) and sulcus	0.73172	0.02061	0.75060	0.03183	2,17	0,69	0,10
lh_paracentral lobule and sulcus	0.73887	0.01895	0.74781	0.02140	1,35	0,44	0,29
lh_subcentral gyrus (central operculum) and sulci	0.69975	0.01263	0.72223	0.01902	4,29	1,37	**0,00****
lh_transverse frontopolar gyri and sulci	0.65609	0.01341	0.66443	0.02949	1,13	0,35	0,39
lh_anterior part of the cingulate gyrus and sulcus (aCC)	0.67624	0.00752	0.68404	0.01794	1,77	0,55	0,18
lh_middle-anterior part of the cingulate gyrus and sulcus (amCC)	0.68864	0.01368	0.69638	0.02080	1,35	0,43	0,29
lh_middle-posterior part of the cingulate gyrus and sulcus (pmCC)	0.70532	0.01936	0.71478	0.01875	1,50	0,50	0,25
lh_posterior-dorsal part of the cingulate gyrus (dpCC)	0.72234	0.01218	0.73379	0.02327	1,91	0,60	0,14

lh_posterior-ventral part of the cingulate gyrus (vpCC, isthmus of the cingulate gyrus)	0.74569	0.01374	0.76949	0.03157	3,05	0,95	**0,02***
lh_Cuneus (o6)	0.73493	0.01627	0.73950	0.02748	0,63	0,20	0,63
lh_opercular part of the inferior frontal gyrus	0.68544	0.01031	0.69852	0.01605	2,99	0,95	**0,02***
lh_orbital part of the inferior frontal gyrus	0.67914	0.01516	0.69354	0.02026	2,47	0,80	0,06
lh_triangular part of the inferior frontal gyrus	0.68052	0.01429	0.69276	0.01924	2,22	0,71	0,09
lh_middle frontal gyrus (F2)	0.66731	0.01050	0.67678	0.01914	1,90	0,60	0,14
lh_superior frontal gyrus (F1)	0.68053	0.01207	0.68817	0.02015	1,42	0,45	0,27
lh_long insular gyrus and central sulcus of the insula	0.67885	0.01392	0.68893	0.01933	1,84	0,59	0,16
lh_short insular gyri	0.65912	0.00910	0.67120	0.01289	3,33	1,07	**0,01***
lh_middle occipital gyrus (o2, lateral occipital gyrus)	0.71172	0.01478	0.71981	0.02051	1,39	0,45	0,28
lh_superior occipital gyrus (o1)	0.72018	0.01756	0.72842	0.02439	1,19	0,38	0,36
lh_lateral occipito-temporal gyrus (fusiform gyrus, o4-t4)	0.71912	0.01527	0.74182	0.02630	3,27	1,03	**0,01***
lh_lingual gyrus, lingual part of the medial occipito-temporal gyrus, (o5)	0.73499	0.01631	0.75101	0.02215	2,53	0,81	0,05
lh_parahippocampal gyrus, parahippocampal part of the medial occipito-temporal gyrus, (t5)	0.69684	0.01554	0.71631	0.01884	3,44	1,12	**0,01***
lh_orbital gyri	0.70025	0.01416	0.72404	0.06246	1,65	0,51	0,21
lh_angular gyrus	0.68392	0.01100	0.69137	0.02183	1,34	0,42	0,30
lh_supramarginal gyrus	0.68789	0.01155	0.69991	0.01954	2,32	0,73	0,08
lh_superior parietal lobule (lateral part of p1)	0.69367	0.01365	0.69986	0.02305	1,01	0,32	0,44
lh_postcentral gyrus	0.70855	0.01742	0.71837	0.03727	1,05	0,33	0,42
lh_precentral gyrus	0.72708	0.01654	0.73803	0.02327	1,67	0,53	0,20
lh_precuneus (medial part of p1)	0.70643	0.00892	0.71291	0.02207	1,20	0,37	0,36
lh_straight gyrus, gyrus rectus	0.68529	0.01046	0.71660	0.08612	1,61	0,49	0,22
lh_subcallosal area, subcallosal gyrus	0.64024	0.07068	0.65983	0.05771	0,91	0,31	0,49
lh_anterior transverse temporal gyrus (of heschl)	0.74462	0.02050	0.74606	0.03618	0,15	0,05	0,90

lh_lateral aspect of the superior temporal gyrus	0.69164	0.01396	0.70398	0.01855	2,31	0,74	0,08
lh_planum polare of the superior temporal gyrus	0.67115	0.01476	0.67365	0.01919	0,45	0,14	0,75
lh_planum temporale or temporal plane of the superior temporal gyrus	0.69971	0.01673	0.69546	0.03571	-0,47	-0,15	0,73
lh_Inferior temporal gyrus (t3)	0.68686	0.01547	0.70696	0.02019	3,42	1,10	**0,01***
lh_middle temporal gyrus (t2)	0.68208	0.01013	0.69139	0.01842	1,94	0,61	0,14
lh_horizontal ramus of the anterior segment of the lateral sulcus (or fissure)	0.70479	0.01340	0.73672	0.02472	4,98	1,57	**0,00****
lh_vertical ramus of the anterior segment of the lateral sulcus (or fissure)	0.70529	0.01737	0.72081	0.01949	2,56	0,84	**0,05***
lh_posterior ramus (or segment) of the lateral sulcus (or fissure)	0.72861	0.01546	0.73730	0.02258	1,38	0,44	0,29
lh_occipital pole	0.73809	0.01756	0.75617	0.02731	2,43	0,77	0,06
lh_temporal pole	0.67477	0.01085	0.69091	0.02469	2,64	0,82	**0,05***
lh_Calcarine sulcus	0.73564	0.01591	0.75364	0.02790	2,45	0,78	0,06
lh_Central sulcus (Rolando's fissure)	0.74204	0.01447	0.74199	0.02512	-0,01	0,00	0,99
lh_marginal sulcus of cingulate gyrus	0.70202	0.01903	0.71128	0.02263	1,35	0,44	0,29
lh_anterior circular sulcus of the insula	0.68486	0.01223	0.70352	0.02043	3,43	1,09	**0,01***
lh_Inferior circular sulcus of the insula	0.68801	0.01227	0.68839	0.02124	0,07	0,02	0,96
lh_superior circular sulcus of the insula	0.70022	0.01002	0.71803	0.01656	4,02	1,28	**0,00****
lh_anterior transverse collateral sulcus	0.68337	0.01702	0.71181	0.03010	3,60	1,14	**0,01****
lh_posterior transverse collateral sulcus	0.74995	0.02451	0.78360	0.04344	2,95	0,93	**0,03***
lh_Inferior frontal sulcus	0.70204	0.01364	0.71391	0.02056	2,10	0,67	0,11
lh_middle frontal sulcus	0.67529	0.01167	0.68273	0.01939	1,44	0,46	0,27
lh_superior frontal sulcus	0.68534	0.01049	0.69944	0.02098	2,64	0,83	**0,05***
lh_Intermediate sulcus of Jensen	0.68899	0.01463	0.70134	0.03523	1,43	0,44	0,27
lh_Intraparietal sulcus and posterior transverse parietal sulcus	0.71367	0.01245	0.72436	0.02153	1,88	0,60	0,15
lh_middle occipital sulcus and sulcus lunatus (secondary intermediate sulcus)	0.71891	0.01489	0.72653	0.02658	1,10	0,35	0,40
lh_superior occipital sulcus and transverse occipital sulcus (superior intermediate sulcus)	0.72340	0.01500	0.72306	0.02322	-0,05	-0,02	0,96

lh_anterior occipital sulcus	0.72081	0.01929	0.73293	0.03427	1,35	0,43	0,29
lh_lateral occipito-temporal sulcus	0.70828	0.01815	0.73391	0.04127	2,51	0,78	0,06
lh_medial occipito-temporal sulcus and lingual sulcus	0.71500	0.01633	0.74723	0.03118	4,02	1,26	**0,00****
lh_lateral orbital sulcus	0.67609	0.01498	0.69003	0.02164	2,30	0,74	0,08
lh_medial orbital sulcus, olfactory sulcus	0.70981	0.01086	0.72771	0.04445	1,74	0,53	0,19
lh_h-shaped orbital sulcus	0.70873	0.01542	0.72628	0.02525	2,59	0,82	**0,05***
lh_parieto-occipital sulcus	0.73912	0.00944	0.75029	0.02257	2,02	0,63	0,12
lh_pericallosal sulcus	0.73907	0.04294	0.77133	0.03272	2,53	0,85	0,05
lh_postcentral sulcus	0.71074	0.01167	0.72508	0.02532	2,27	0,71	0,08
lh_Inferior part of the precentral sulcus	0.70024	0.00830	0.70517	0.02113	0,96	0,30	0,47
lh_superior part of the precentral sulcus	0.71419	0.01214	0.72054	0.02379	1,05	0,33	0,43
lh_suborbital sulcus	0.68078	0.00987	0.69393	0.02847	1,93	0,60	0,14
lh_subparietal sulcus	0.71819	0.01133	0.72865	0.02287	1,80	0,57	0,17
lh_Inferior temporal sulcus	0.69451	0.01279	0.72020	0.02676	3,81	1,19	**0,01***
lh_superior temporal sulcus	0.70438	0.01160	0.71669	0.01708	2,60	0,83	**0,05***
lh_transverse temporal sulcus (heschl's gyrus)	0.73535	0.02174	0.73684	0.03171	0,17	0,05	0,89
rh_unknown	0.16848	0.01452	0.16215	0.01653	-1,24	-0,40	0,34
rh_Fronto-marginal gyrus (of Wernicke) and sulcus	0.67384	0.01283	0.69396	0.09622	0,93	0,28	0,49
rh_Inferior occipital gyrus (o3) and sulcus	0.74189	0.02307	0.75360	0.02943	1,36	0,44	0,29
rh_paracentral lobule and sulcus	0.73958	0.01315	0.75734	0.02388	2,85	0,90	**0,03***
rh_subcentral gyrus (central operculum) and sulci	0.71811	0.01664	0.72735	0.02032	1,52	0,49	0,24
rh_transverse frontopolar gyri and sulci	0.65464	0.01149	0.65782	0.03287	0,41	0,13	0,77
rh_anterior part of the cingulate gyrus and sulcus (aCC)	0.67273	0.00965	0.69117	0.01703	4,12	1,30	**0,00****
rh_middle-anterior part of the cingulate gyrus and sulcus (amCC)	0.69436	0.01058	0.71035	0.01507	3,78	1,21	**0,01****
rh_middle-posterior part of the cingulate gyrus and sulcus (pmCC)	0.72020	0.00864	0.73342	0.01625	3,15	0,99	**0,02***

rh_posterior-dorsal part of the cingulate gyrus (dpCC)	0.73079	0.01021	0.74337	0.01731	2,74	0,87	**0,04***
rh_posterior-ventral part of the cingulate gyrus (vpCC, isthmus of the cingulate gyrus)	0.75964	0.03107	0.78148	0.03021	2,16	0,71	0,10
rh_Cuneus (o6)	0.74314	0.01774	0.74530	0.02309	0,32	0,10	0,81
rh_opercular part of the inferior frontal gyrus	0.69359	0.01080	0.70254	0.01709	1,93	0,61	0,14
rh_orbital part of the inferior frontal gyrus	0.68158	0.01517	0.69237	0.01945	1,89	0,61	0,14
rh_triangular part of the inferior frontal gyrus	0.68662	0.01307	0.68880	0.01702	0,44	0,14	0,75
rh_middle frontal gyrus (F2)	0.67098	0.01011	0.67895	0.01605	1,83	0,58	0,16
rh_superior frontal gyrus (F1)	0.67681	0.00944	0.69216	0.01801	3,31	1,04	**0,01***
rh_long insular gyrus and central sulcus of the insula	0.67021	0.01161	0.67555	0.01220	1,36	0,45	0,29
rh_short insular gyri	0.67135	0.01202	0.67851	0.01449	1,64	0,53	0,20
rh_middle occipital gyrus (o2, lateral occipital gyrus)	0.71315	0.00980	0.72405	0.02078	2,09	0,65	0,11
rh_superior occipital gyrus (o1)	0.72724	0.01351	0.73296	0.02014	1,03	0,33	0,43
rh_lateral occipito-temporal gyrus (fusiform gyrus, o4-t4)	0.75536	0.01412	0.76629	0.02346	1,74	0,55	0,18
rh_lingual gyrus, lingual part of the medial occipito-temporal gyrus, (o5)	0.75218	0.01610	0.75843	0.02396	0,94	0,30	0,47
rh_parahippocampal gyrus, parahippocampal part of the medial occipito-temporal gyrus, (t5)	0.71802	0.01294	0.72893	0.01950	2,03	0,65	0,12
rh_orbital gyri	0.68616	0.01083	0.70892	0.09492	1,06	0,32	0,42
rh_angular gyrus	0.69831	0.01049	0.70178	0.01639	0,78	0,25	0,55
rh_supramarginal gyrus	0.70146	0.01201	0.70429	0.01679	0,59	0,19	0,66
rh_superior parietal lobule (lateral part of p1)	0.70247	0.01059	0.70309	0.02679	0,10	0,03	0,94
rh_postcentral gyrus	0.72389	0.01964	0.72738	0.02147	0,52	0,17	0,71
rh_precentral gyrus	0.73838	0.01343	0.75160	0.02218	2,23	0,71	0,09
rh_precuneus (medial part of p1)	0.71568	0.01156	0.72574	0.02087	1,85	0,58	0,16
rh_straight gyrus, gyrus rectus	0.69134	0.01166	0.72725	0.12620	1,27	0,38	0,34
rh_subcallosal area, subcallosal gyrus	0.58262	0.10558	0.60313	0.08010	0,66	0,22	0,62

rh_anterior transverse temporal gyrus (of heschl)	0.75789	0.02135	0.77162	0.02578	1,77	0,58	0,17
rh_lateral aspect of the superior temporal gyrus	0.70751	0.01491	0.71666	0.01855	1,66	0,54	0,20
rh_planum polare of the superior temporal gyrus	0.67672	0.01520	0.69178	0.01767	2,79	0,91	**0,03***
rh_planum temporale or temporal plane of the superior temporal gyrus	0.71839	0.02103	0.72626	0.01950	1,17	0,39	0,37
rh_Inferior temporal gyrus (t3)	0.71624	0.01318	0.72257	0.02191	1,08	0,34	0,41
rh_middle temporal gyrus (t2)	0.68529	0.01173	0.69596	0.01609	2,33	0,75	0,08
rh_horizontal ramus of the anterior segment of the lateral sulcus (or fissure)	0.71695	0.01453	0.73032	0.02194	2,21	0,71	0,09
rh_vertical ramus of the anterior segment of the lateral sulcus (or fissure)	0.71442	0.01927	0.71179	0.02542	-0,36	-0,11	0,80
rh_posterior ramus (or segment) of the lateral sulcus (or fissure)	0.73656	0.01146	0.74135	0.02157	0,86	0,27	0,51
rh_occipital pole	0.74634	0.01695	0.75539	0.02480	1,31	0,42	0,31
rh_temporal pole	0.68972	0.01449	0.70247	0.01880	2,33	0,75	0,08
rh_Calcarine sulcus	0.75094	0.01597	0.76116	0.02277	1,60	0,51	0,22
rh_Central sulcus (Rolando's fissure)	0.74783	0.01465	0.75165	0.02073	0,65	0,21	0,62
rh_marginal sulcus of cingulate gyrus	0.71986	0.01179	0.72585	0.01649	1,28	0,41	0,32
rh_anterior circular sulcus of the insula	0.68516	0.01188	0.69436	0.01624	1,98	0,64	0,13
rh_Inferior circular sulcus of the insula	0.69528	0.01314	0.70520	0.01790	1,94	0,62	0,14
rh_superior circular sulcus of the insula	0.70005	0.01248	0.70711	0.01777	1,41	0,45	0,27
rh_anterior transverse collateral sulcus	0.73145	0.01724	0.74155	0.02332	1,51	0,49	0,25
rh_posterior transverse collateral sulcus	0.77674	0.02738	0.78881	0.03453	1,19	0,38	0,36
rh_Inferior frontal sulcus	0.71042	0.01584	0.71438	0.02166	0,64	0,21	0,63
rh_middle frontal sulcus	0.67742	0.01065	0.67907	0.01919	0,33	0,10	0,81
rh_superior frontal sulcus	0.68884	0.01059	0.69988	0.02173	2,01	0,63	0,12
rh_Intermediate sulcus of Jensen	0.70200	0.01995	0.70622	0.02603	0,56	0,18	0,68
rh_Intraparietal sulcus and posterior transverse parietal sulcus	0.72643	0.01074	0.72888	0.02185	0,44	0,14	0,75
rh_middle occipital sulcus and sulcus lunatus (secondary intermediate sulcus)	0.72983	0.01803	0.73745	0.02498	1,07	0,35	0,41

rh_superior occipital sulcus and transverse occipital sulcus (superior intermediate sulcus)	0.73618	0.01222	0.73493	0.01940	-0,24	-0,08	0,85
rh_anterior occipital sulcus	0.73519	0.01608	0.74327	0.02560	1,17	0,37	0,37
rh_lateral occipito-temporal sulcus	0.75357	0.01880	0.76357	0.03393	1,13	0,36	0,39
rh_medial occipito-temporal sulcus and lingual sulcus	0.74359	0.01648	0.76287	0.02685	2,67	0,85	**0,04***
rh_lateral orbital sulcus	0.67978	0.01529	0.68415	0.01875	0,78	0,25	0,55
rh_medial orbital sulcus, olfactory sulcus	0.68782	0.01620	0.70622	0.06759	1,18	0,36	0,37
rh_h-shaped orbital sulcus	0.69404	0.01155	0.70674	0.04108	1,32	0,41	0,31
rh_parieto-occipital sulcus	0.75702	0.01224	0.76202	0.01950	0,95	0,30	0,47
rh_pericallosal sulcus	0.71968	0.06646	0.77529	0.02617	3,24	1,14	**0,02***
rh_postcentral sulcus	0.72898	0.01801	0.73765	0.02105	1,35	0,44	0,29
rh_Inferior part of the precentral sulcus	0.70414	0.01224	0.71447	0.01850	2,03	0,65	0,12
rh_superior part of the precentral sulcus	0.71716	0.01145	0.72095	0.02208	0,67	0,21	0,62
rh_suborbital sulcus	0.67372	0.01091	0.69935	0.05341	2,09	0,64	0,12
rh_subparietal sulcus	0.72403	0.00912	0.73569	0.01985	2,35	0,73	0,08
rh_Inferior temporal sulcus	0.70945	0.01508	0.72246	0.02317	2,05	0,65	0,12
rh_superior temporal sulcus	0.71848	0.01096	0.72620	0.01660	1,69	0,54	0,19
rh_transverse temporal sulcus (heschl's gyrus)	0.74698	0.01477	0.76004	0.02253	2,11	0,67	0,11

p < ,05, **p < 0,01, ***p < ,001; MS = multiple sclerosis HC = healthy controls; NMOSD = neuromyelitis optica spectrum disorders; lh = left hemisphere; rh = right hemisphere.

Supplementary Table 5

Pairwise correlations between NDI in white matter fibres not traversing through white matter lesions and the number of cortical lesions in MS

Variable1	Variable 2	Rho	n	p-value (FDR corrected)
CL	LIT_AF_left	-0,68	20	**0,00****
CL	LIT_AF_right	-0,80	20	**0,00*****
CL	LIT_ATR_left	-0,55	20	**0,02***
CL	LIT_ATR_right	-0,57	20	**0,01***
CL	LIT_CC_1	-0,81	20	**0,00*****
CL	LIT_CC_2	-0,84	20	**0,00*****
CL	LIT_CC_3	-0,84	18	**0,00*****
CL	LIT_CC_4	-0,55	15	**0,04***
CL	LIT_CC_5	-0,56	18	**0,02***
CL	LIT_CC_6	-0,73	20	**0,00*****
CL	LIT_CC_7	-0,70	18	**0,00****
CL	LIT_CG_left	-0,84	20	**0,00*****
CL	LIT_CG_right	-0,78	20	**0,00*****
CL	LIT_CST_left	-0,58	20	**0,01***
CL	LIT_CST_right	-0,50	20	**0,04***
CL	LIT_FPT_left	-0,74	20	**0,00*****
CL	LIT_FPT_right	-0,67	20	**0,00****
CL	LIT_ICP_left	-0,33	20	0,18
CL	LIT_ICP_right	-0,09	20	0,72
CL	LIT_IFO_left	-0,28	17	0,30
CL	LIT_IFO_right	-0,56	18	**0,02***
CL	LIT_ILF_left	-0,57	19	**0,02***
CL	LIT_ILF_right	-0,52	17	**0,04***
CL	LIT_MCP_BI	-0,21	20	0,38
CL	LIT_OR_left	-0,35	15	0,23
CL	LIT_OR_right	-0,57	14	**0,04***
CL	LIT_POPT_left	-0,44	20	0,07
CL	LIT_POPT_right	-0,24	19	0,33
CL	LIT_SCP_left	-0,28	20	0,25
CL	LIT_SCP_right	0,16	20	0,51
CL	LIT_SLF_III_left	-0,68	20	**0,00****
CL	LIT_SLF_III_right	-0,80	20	**0,00*****
CL	LIT_SLF_II_left	-0,65	19	**0,01***
CL	LIT_SLF_II_right	-0,77	20	**0,00*****

CL	LIT_SLF_I_left	-0,83	19	**0,00***
CL	LIT_SLF_I_right	-0,72	19	**0,00**
CL	LIT_STR_left	-0,67	18	**0,01**
CL	LIT_STR_right	-0,63	19	**0,01**
CL	LIT_ST_FO_left	-0,68	20	**0,00**
CL	LIT_ST_FO_right	-0,63	20	**0,01**
CL	LIT_ST_PREM_left	-0,76	20	**0,00***
CL	LIT_ST_PREM_right	-0,74	20	**0,00***
CL	LIT_T_OCC_left	-0,35	16	0,21
CL	LIT_T_OCC_right	-0,61	14	**0,03***
CL	LIT_T_PAR_left	-0,54	20	**0,02***
CL	LIT_T_PAR_right	-0,34	19	0,18
CL	LIT_T_PREM_left	-0,66	20	**0,00**
CL	LIT_T_PREM_right	-0,71	20	**0,00**
CL	LIT_UF_left	-0,59	20	**0,01***
CL	LIT_UF_right	-0,65	20	**0,00**

*p < ,05, **p < 0,01, ***p < 0,001; MS,= multiple sclerosis; NDI = neurite density index; CL= cortical lesions count; AF = arcuate fascicle; ATR = anterior thalamic radiation; CC = corpus callosum; CC_1 = rostrum; CC_2 = genu; CC_3 = rostral body; CC_4 = anterior midbody; CC_5 = posterior midbody; CC_6 = isthmus; CC_7 = splenium; CG = cingulum; CST = corticospinal tract; FPT = fronto-pontine tract; ICP = inferior cerebellar peduncle; IFO = inferior occipito-frontal fascicle; ILF = inferior longitudinal fascicle; MCP = middle cerebellar peduncle; OR = optic radiation; POPT = parieto-occipital pontine tract; SCP = superior cerebellar peduncle; SLF_I = superior longitudinal fascicle I; SLF_II = superior longitudinal fascicle II; SLF_III = superior longitudinal fascicle III; STR = superior thalamic radiation; ST_FO = striato-fronto-orbital; ST_PREM = striato-premotor; T_OCC = thalamo-occipital; T_PAR = thalamo-parietal; T_PREM = thalamo-premotor; UF = uncinate fascicle.
